# Pro-angiogenic Ginsenosides F1 and Rh1 Inhibit Vascular Leakage by Modulating NR4A1

**DOI:** 10.1038/s41598-019-41115-2

**Published:** 2019-03-14

**Authors:** Ji In Kang, Yoonjung Choi, Chang-Hau Cui, Daeyoup Lee, Sun Chang Kim, Ho Min Kim

**Affiliations:** 1 Biomedical Science and Engineering Interdisciplinary Program, Korea Advanced Institute of Science and Technology (KAIST), 291 Daehak-ro, Yuseong-gu, Daejeon, 34141 Korea; 2 Department of Biological Sciences, Korea Advanced Institute of Science and Technology (KAIST), 291 Daehak-ro, Yuseong-gu, Daejeon, 34141 Korea; 3 Intelligent Synthetic Biology Center, 291 Daehak-ro, Yuseong-gu, Daejeon, 34141 Korea; 4 Center for Biomolecular & Cellular Structure, Institute for Basic Science (IBS), Daejeon, 34126 Korea; 5 Graduate School of Medical Science & Engineering, Korea Advanced Institute of Science and Technology (KAIST), 291 Daehak-ro, Yuseong-gu, Daejeon, 34141 Korea

## Abstract

Vascular endothelial growth factor (VEGF) plays a key role in angiogenesis, but VEGF-induced angiogenesis is often accompanied by a vascular permeability response. Ginsenosides are triterpenoid saponins from the well-known medicinal plant, ginseng, and have been considered a candidate for modulating angiogenesis. Here, we systemically investigated the effects of 10 different ginsenosides on human umbilical vein endothelial cells and newly identified that two PPT-type ginsenosides, F1 and Rh1 induce the migration and proliferation of endothelial cells. Interestingly, RNA transcriptome analysis showed that gene regulation induced by VEGF in endothelial cells is distinct from that of ginsenoside F1 and Rh1. In addition, F1 and Rh1 significantly inhibited vascular leakage both *in vitro* and *in vivo*, which are induced by vascular endothelial growth factor. Furthermore, comparative transcriptome analysis revealed that these effects of F1 and Rh1 on vascular leakage restoration are mainly caused by changes in VEGF-mediated TNFα signaling via NFκB, particularly by the suppression of expression and transcriptional activity of NR4A1 by F1 and Rh1, even in the presence of VEGF. These findings demonstrate that ginsenosides F1 and Rh1 can be a promising herbal remedy for vessel normalization in ischemic disease and cancer and that NR4A1 is the key target.

## Introduction

Angiogenesis is the process of new blood vessel formation from pre-existing vessels, and is essential for development, reproduction and repair^1^. Physiological angiogenesis is highly complex and requires coordinated processes that are orchestrated by various angiogenic factors, including vascular endothelial growth factor (VEGF), angiopoietins, platelet-derived growth factor (PDGF), transforming growth factor beta (TGF-β), fibroblast growth factor (FGF) and chemokines. Abnormal regulation of angiogenesis has been implicated in the pathogenesis of numerous diseases such as ischemic disease, neurodegeneration, inflammatory disorders, ocular neovascularization, and cancer^2,3^.

Ginsenosides are the molecular components of Araliaceae *Panax* genus plants such as *P. ginseng, P. notoginseng and P. quinquefolius*, and have a wide range of pharmacological effects, including angiogenesis, immunomodulatory, anti-cancer, anti-fatigue, anti-aging, anti-diabetic, antidepressant-like and neuroprotective effects^4^. More than 150 naturally occurring ginsenosides have been identified and most are part of the dammarane family, which are further classified into protopanaxatriols-type (PPT) and protopanaxadiols-type (PPD) ginsenosides^4,5^. Interestingly, depending on the type, different therapeutic effects in cancer, diabetes mellitus, cardiovascular, immune, and central nervous system effects have been reported^4^. In particular, PPT-type ginsenosides (Rg1 and Re)^6,7^ and PPD-type ginsenosides (Rg3, Rb1, Rb2 and CK)^8–11^ have pro-angiogenic and anti-angiogenic effects, respectively, even though both PPT-type and PPD-type ginsenosides consist of the same steroid-like backbone, except the position and number of attached sugar moieties (Supplementary Fig. 1A,B).

VEGF are arguably the most widely studied class of angiogenic factors. VEGF binding to VEGF receptors promotes the migration and proliferation of vascular endothelial cells, which are key features that are required for blood vessel development^12^. In addition, VEGF potently induces vascular leakage by forming endothelial vesiculovacuolar organelles (VVOs) and fenestrations, or by disrupting the junctional integrity between vascular endothelial cells, which are maintained by junctional proteins such as VE-cadherin, occludin, ZO-1, and claudin^13^. Although paracrine activation of endothelial cells by VEGF is a major initiating event in angiogenesis, deregulated expression of VEGF and subsequently induced vascular leakage can also promote edema and extensive tissue injury in ischemic disease and tumor cell extravasation, and metastasis in cancer^13,14^. Recent studies have shown that altering angiogenesis through the Tie2 pathway or reducing glycolysis in tumor endothelial cells can normalize tumor vasculature, thus enhancing blood perfusion and drug delivery, and reducing tumor growth and metastasis^15,16^. Therefore, effective blockage of VEGF-induced vascular leakage in pathologic conditions could have substantial therapeutic benefits. Interestingly, unlike VEGF, several pro-angiogenic factors such as angiopoietin-1 (ANG1), fibroblast growth factor (FGF) and hepatocyte growth factor (HGF) enhance endothelial junctional integrity and vascular barrier function, and can even block VEGF-induced vascular leakage^17–19^. Although PPD-type ginsenosides, Rb1 and Rk1, have been reported to inhibit vascular permeability induced by VEGF, lipopolysaccharide, thrombin, or histamine^20,21^, it is not yet clear whether pro-angiogenic ginsenosides can induce vascular leakage similar to VEGF, or act as a leakage inhibitor, similar to ANG1, FGF and HGF.

In this study, we systemically investigated the angiogenic activities of 10 ginsenoside variants by tube formation, migration and proliferation assays in human umbilical vein endothelial cells (HUVECs), identifying F1 and Rh1 as novel pro-angiogenic ginsenosides. Whole transcriptome analysis indicated that gene expression profiles upon F1 or Rh1 treatment in HUVECs are largely different from those in VEGF treatment. Moreover, we revealed that F1 and Rh1 do not induce vascular leakage and can even inhibit VEGF-induced vascular leakage *in vitro* and *in vivo* by suppressing NR4A1’s transcriptional activity as well as decreasing the gene expression of NR4A1, which mediates acute and chronic vascular hyperpermeability^22^.

## Materials and Methods

### Production of Single Ginsenoside Variants

Minor ginsenosides including F1, F2, Rh1(S), Rh2(S), Rg3(S), and CK (>95% pure) were prepared using enzymatic methods previously reported^23–28^. Briefly, PPT type (Daziran Co. Ltd.) or PPD type (Hongjiou Biotech Co. Ltd.) major ginsenosides mixtures were converted into minor ginsenosides using various recombinant β-glucosidases and the produced minor ginsenosides were purified using a silica column (168 × 71 mm id, Biotage, Sweden) and ODS column (157 × 39 mm id, Biotage, Sweden). They were then further purified by Recycling-Preparative HPLC (Japan Analytical Industry Co. Ltd.) with a JAIGEL-ODS-AP column (10 μm, 500 × 20 mm id, Japan Analytical Industry Co., Ltd.). Major ginsenosides including Rg1, Re, Rb1, and Rd were directly purified from PPT- or PPD-type major ginsenoside mixtures using a silica column, ODS column and Recycling-Preparative HPLC. The compounds were dissolved in 100% dimethyl sulfoxide (DMSO) and diluted with the medium for the sample preparation.

### Cell Culture

Human umbilical vein endothelial cells (HUVECs, Cat#CC-2519, Lonza), human retinal microvascular endothelial cells (HRMECs, Cat#ACBRI 181, Cell Systems) and human embryonic kidney cells 293 T (HEK293T, Cat#CRL-3216, ATCC) were authenticated according to ATCC guidelines and used within 6 months of receipt. HUVECs and HRMECs were cultured in EBM-2 (Cat#CC-3156, Lonza) supplemented with EGM-2 (Cat#CC-3162, Lonza) and 100 µg/ml penicillin/streptomycin on gelatin (Cat#G1890, Sigma-Aldrich; 0.1% in DDW) pre-coated plates. HEK293T were cultured in DMEM (Cat#LM001-5, Welgene) supplemented with 10% FBS (S001-01, Welgene) and 100 µg/ml antibiotics-antimycotics. All cells were grown at 37 °C and 5% CO_2_. All experiments have been performed in accordance with the institutional guidelines.

### Tube Formation Assay

Either HUVECs or HRMECs were plated at 6,000 cells/well in EBM-2 medium containing 0.1% FBS on Matrigel-coated-96 well plates (Cat# 354230, Corning) and were treated with the indicated concentrations of ginsenosides or 0.5 nM VEGF^29,30^. After a 4 hr incubation, tube formation was observed with a cell analyzer (JuLITM, Cat# JULI-B004, NanoEnTek). Tubes forming intact networks were quantified by counting the number of branch points of the capillary-like tubes from 5 random fields/well in a blinded manner, under an inverted microscope.

### Cell Proliferation Assay

Cell proliferation was determined with a WST-1 assay^29,30^. Briefly, HUVECs or HRMECs were seeded at 3,000 cells/well on 96-well plates with indicated concentrations of ginsenosides (3, 6, and 12 μM). After 24 hr, WST-1 (water-soluble, tetrazolium salt, Cat# EZ-1000, DOGEN) was added (1:10 final dilution) and the cells were cultured for additional 4 hr. The absorbance was then measured at 450 nm with a microplate reader (TriStar² LB 942, Berthold).

### Cell Migration Assay

HUVECs (80,000 cells/well) or HRMECs (40,000 cells/well) were seeded and cultured on the culture-inserts of μ-dishes (Cat# 81176, Ibidi) until reaching confluence. The culture-inserts were subsequently removed to generate wound gaps. Fresh EBM-2 medium (supplemented with 0.1% FBS) was added with 2.5 nM VEGF^29,30^ or the indicated concentrations of ginsenosides. After 12 hr and 24 hr, the migrated cells within the wound were monitored with a cell analyzer, JuLITM (Cat#JULI-B004, NanoEnTek). Cell migration was quantified by measuring the ratio of the migration area to the total area of the wound gap using ImageJ software (NIH).

### mRNA-sequencing and Data Analysis

mRNA was extracted from HUVECs treated with VEGF (2.5 nM) or ginsenosides (F1 and Rh1, 10 μM) for 1 hr using a Magnetic mRNA Isolation Kit (NEB) according to the manufacturer’s protocol. The DMSO-treated HUVECs were used as a control. DNase-treated mRNA was subjected to library preparation using a NEXTflex™ Rapid Directional mRNA-Seq kit (BIOO) according to the commercially available protocols. Enriched libraries were sequenced on a HiSeq. 2500 (illumina) using the single-end method (50-bp reads). The sequenced reads were aligned to the human genome (version: Hg19) with the STAR software (v.2.4.0), using default parameters^31^. For each gene, the reads per kilobase per million (RPKM) was calculated using the HOMER anlayzeReapeats tool with the “-rpkm” option^32^. DEGs were identified using the DESeq package in Bioconductor^33^. Heatmaps were visualized by R statistical programming language v.3.3.0. (http://www.r-project.org/) with the pheatmap function. The GO analysis for up- and down-regulated genes in VEGF and ginsenosides-treated cells was carried out by ConsensusPathDB database (http://consensuspathdb.org/). The significance threshold was defined by a p-value of less than 0.01. Data were deposited in the GEO repository under accession number GSE116121.

### *In vitro* Cell Permeability Assay

The *in vitro* permeability assay was performed with the *In Vitro* Vascular Permeability Assay Kit (Cat#ECM644, Millipore)^34^. In brief, HUVECs and HRMECs were seeded into the insert plate and then incubated for 72 hr to form a monolayer. The cell monolayers were then treated with VEGF (2.5 nM) or indicated ginsenosides (10 μM). Otherwise, they were pre-treated with VEGF (2.5 nM) for an hour and sequentially treated with indicated ginsenosides (10 μM) for another hour. Then, a high molecular weight FITC-Dextran was added on top of the cells, allowing the fluorescent molecules to pass through the cell monolayer, and incubated for 20 min at room temperature in the dark. The extent of permeability was determined by measuring the fluorescence of the receiver plate well solution using a fluorescence plate reader with 485 nm excitation and 535 nm emission filters.

### *In vivo* Vascular Permeability Assay

Animal care and experimental procedures were performed under the approval from the Animal Care Committees of KAIST. Vascular permeability was quantified using the modified Miles assay^17,35^. Evans blue dye (Cat#E2129, Sigma Aldrich) was injected intravenously into the anesthetized mice. After 10 min, DMSO (0.01% in 10 μl of PBS, negative control), VEGF alone (250 ng, positive control), indicated ginsenosides (10 μM) alone, or indicated ginsenosides (10 μM) together with VEGF (250 ng) in 10 μl of PBS was intradermally injected into the ear. After 30 min, the ears were collected and incubated with formamide (Cat#F9037, Sigma Aldrich) at 56 °C for 48 hr. The concentration of Evans blue in the ears and standards were determined by reading the optical density at 620 nm with a spectrophotometer.

### Gene Set Enrichment Analysis (GSEA)

GSEA^36^ was performed using GSEA software (version 3.0) with 1,000 phenotype permutations and default values for other parameters. Gene sets used in this study were selected from the MSigDB hallmark gene sets (http://software.broadinstitute.org/gsea/msigdb/collections.jsp).

### Quantitative real-time PCR

Quantitative real-time PCR was performed using the iQ SYBR Green Supermix (Cat#1709990, Bio-Rad) and the CFX-96 real-time PCR detection system (Bio-Rad) with primer pairs listed in Supplementary Table 3. The relative levels of each mRNA expression were calculated using the ΔΔC_t_ method normalized to the level of β-actin mRNA expression in the same sample with CFX manager soft-ware (Bio-Rad).

### Western blot analysis

HUVECs were treated with VEGF (2.5 nM) and ginsenosides (F1 or Rh1, 10 μM) for an hour. Otherwise, HUVECs were pre-treated with VEGF (2.5 nM) for an hour and treated with ginsenosides (F1 or Rh1, 10 μM) for another hour. The cells were lysed in the RIPA buffer (Cat#BRI-9001, T&I) including the protease inhibitor (Cat#11 836 170 001, Roche) and phosphatase inhibitor (Cat#04 906 845 001, Roche). Equivalent amounts of protein per sample (40 μg) were resolved on 10% SDS-PAGE and transferred to polyvinylidene difluoride (PVDF) membranes. NR4A1 was detected with anti-NR4A1 antibody (1: 100 dilution; Cat#sc-365113, Santa Cruz) and the anti-β-actin antibody (1:1000 dilution; Cat#ab8227, Abcam) was used to normalize the loading cell lysates. Other antibodies for this study are listed in Supplementary Table 4.

### NR4A1 Reporter Gene Assay

HEK293T cells were transfected with the NR4A1 response element (NurRE) luciferase reporter construct which contains 3 copies of NR4A1 binding sites from the POMC gene promoter^37^, and 10 μM of ginsenosides (F1 or Rh1) were treated for 1 hr. For the positive control (stimulation of transcription activity of NR4A1), 0.1 μM of Csn-B (Cat#C2997, Sigma-Aldrich), which is a fungal metabolite closely related to phomposin C and the naturally occurring agonist for NR4A1 with high affinity (IC_50_ = 0.278 nM)^38^, was co-treated. Then, the dual luciferase reporter assay (Cat#E1910, Promega) was performed by following the manufacturer’s instruction.

### Immunofluorescence staining

HUVECs were grown to confluence on 8-well cell culture slides (Cat#30108, SPL). The cells were pre-treated with VEGF (2.5 nM) for an hour and treated with ginsenosides (F1 or Rh1, 10 μM) for another hour. Otherwise, the cells were co-treated with Csn-B (1 μM) and ginsenosides (F1 or Rh1, 10 μM) for an hour. After the treatment, the cells were washed with PBS and were fixed in 4% paraformaldehyde (Cat#P2031, Biosesang) for 20 min at room temperature. The slides were blocked in 1% bovine serum albumin (BSA) diluted in PBST (0.1% Tween 20 in PBS) for 30 min at room temperature. Then, they were incubated with anti-VE-cadherin primary antibody (1:50 dilution; Cat#sc-9989, Santa Cruz) at 4 °C overnight followed by FITC-conjugated secondary antibody (1:50 dilution; Cat#sc-516140, Santa Cruz). Nuclei were counterstained with 0.5 μg/ml of DAPI (Cat#D9542, Sigma Aldrich) and the cells were mounted with permanent aqueous mounting medium (Cat#M01, Biomeda). Immunofluorescence was visualized by confocal microscopy (LSM 780, Zeiss).

### Statistical analysis

All data are presented as the mean ± standard deviation (SD) of experiments performed in triplicate. Significant differences were analyzed by one-way ANOVA and Turkey’s multiple comparisons test (Graphpad Prism software). Differences were considered significant when P < 0.05 (*P < 0.05; **P < 0.01; ***P < 0.001).

## Results

### F1 and Rh1 promote tube formation, proliferation and migration of endothelial cell

**Figure 1:**
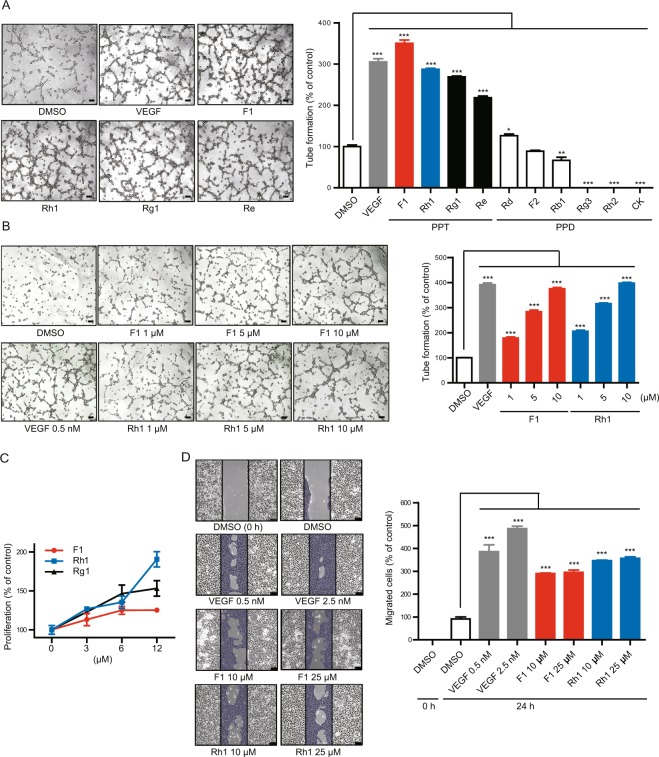
F1 and Rh1 promote endothelial cell tube formation, proliferation and migration. (**A**) Effects of 10 different ginsenosides on tube formation. The tube formation assay was performed using HUVECs with 4 hr DMSO treatment as control, VEGF (0.5 nM), or indicated ginsenosides (25 μM). Representative tube formation images of PPT-type ginsenosides, which have pro-angiogenic effects, are shown (left). Scale bars, 100 μm. The branch points of the capillary-like tubes were counted and the quantitative data are presented as mean ±SD (n = 3) (right). (**B**) Dose-dependent effects of F1 and Rh1 on tube formation in HUVECs. Representative images (left) and quantification (right) of the tube formation assay are shown. Scale bars, 100 μm. Data are presented as mean ±SD (n = 3). (**C**) Effects of F1 and Rh1 on cell proliferation. HUVECs were treated with the indicated concentrations of F1, Rh1, or Rg1 for 24 hr and cell proliferation was measured by the WST-1 assay. (**D**) Effects of F1 and Rh1 on cell migration. The cell migration assay was conducted in HUVECs treated with VEGF (0.5 nM or 2.5 nM) or indicated ginsenosides (10 μM or 25 μM) for 12 hr. Representative cell migration images are shown and the wound-healing areas are indicated in blue (left). Scale bars, 100 μm. The migration areas were measured by ImageJ software and the quantitative data are presented as mean ± SD (n = 3) (right). Statistical significance was calculated based on three independent experiments (*P < 0.05; **P < 0.01; ***P < 0.001, P-values between depicted groups).

We used recombinant glucoside hydrolases to produce the minor ginsenosides, including F1, F2, Rh1, Rh2, Rg3, and CK from the crude PPT or PPD mixture in a gram-scale and high purity (>95%) (Supplementary Fig. 1). We also isolated the major ginsenosides including Rg1, Re, Rb1, and Rd from the PPT or PPD mixture (Supplementary Fig. 1). We systemically investigated the angiogenic effects of these variants using a tube formation assay with HUVECs. The results indicated that Rg1 and Re could induce tube formation, while CK and Rg3 inhibit tube formation, which is consistent with previous reports^6–8,11^ (Fig. 1A and Supplementary Fig. 2A). Notably, we determined that F1 and Rh1 could promote tube formation to levels that were similar to that of VEGF and Rg1. Moreover, we found that F1 and Rh1 promote tube formation of HUVECs, as well as human retinal microvascular endothelial cells (HRMECs), in a dose-dependent manner (Fig. 1B and Supplementary Fig. 2B). To further investigate the pro-angiogenic effects of F1 and Rh1, we assessed the effect on the proliferation and migration of endothelial cells upon treatment with F1 or Rh1 in both HUVECs and HRMECs. Similar to VEGF, F1 and Rh1 strongly induced the proliferation and migration of both HUVECs and HRMECs, (Fig. 1C,D, Supplementary Figs 2C,D and 3). These results indicate that ginsenoside variants F1 and Rh1 effectively stimulate angiogenesis *in vitro*.

### F1- and Rh1-mediated angiogenesis are largely different from VEGF-mediated angiogenesis

Because F1 and Rh1 can induce tube formation, proliferation, and migration of endothelial cells similar to VEGF, we investigated whether the molecular mechanism of F1 and Rh1-mediated angiogenesis is similar to that of VEGF. To test this, we performed RNA-sequencing after an hour treatment with VEGF (2.5 nM) or ginsenosides (F1 or Rh1, 10 μM) to HUVECs, and analyzed their gene-expression profiles. We first determined the differential expressed genes (DEGs) in VEGF, F1 and Rh1 treatments versus the DMSO control (Fold change |log_2_| ≥0.5, P-value < 0.1) (Supplementary Fig. 4, and Table 1), and compared these selected DEGs. Surprisingly, only ~20% of up-regulated genes (30/146) and 30% of down-regulated genes (45/150) in VEGF-treated cells were commonly regulated by ginsenosides (F1 or Rh1).

**Figure 2:**
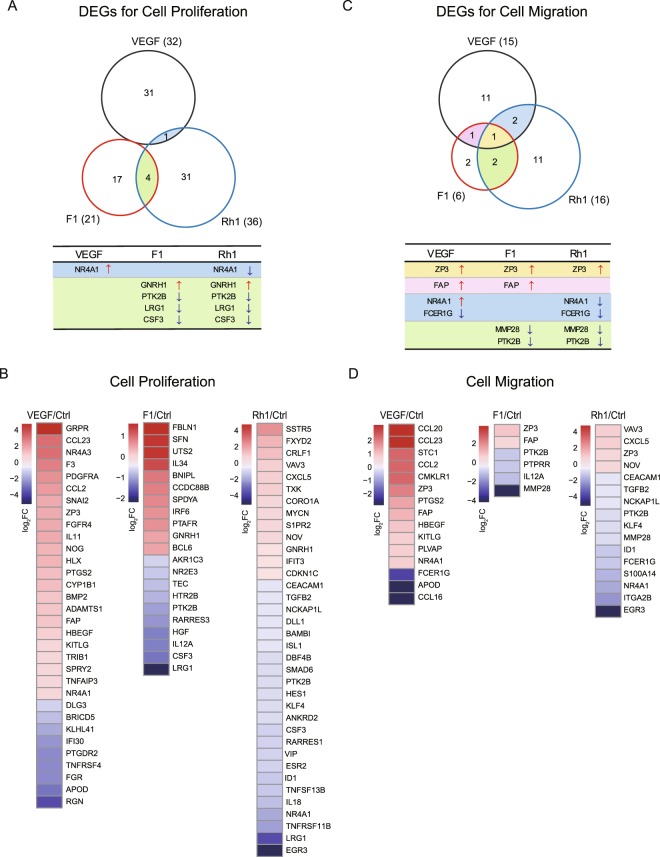
F1 and Rh1 regulate expression of angiogenesis-related genes differently from VEGF. (**A**,**C**) Venn diagrams show the overlapping subsets of differentially expressed genes (DEGs) for cell proliferation **(A)** and for cell migration-related genes **(C)** in VEGF, F1 and Rh1 treatment versus DMSO-treated control sample (Fold change |log_2_| ≥0.5, P-value < 0.1). Overlapping DEGs list are indicated in the table below with the same color as the Venn diagram intersections in A and C. (**B**,**D**) Heatmap representation showing gene expression changes of DEG subsets for cell proliferation (**B**) and for cell migration-related genes (**D**) in VEGF, F1 or Rh1 treatment group versus DMSO control group. Expression levels were shown as relative values (log_2_) normalized to DMSO-treated control sample. Red, up-regulated; blue, down-regulated.

Because F1 and Rh1 can induce HUVECs and HRMECs proliferation, similar to VEGF, we compiled a list of the cell proliferation-related genes from the KEGG database (http://www.genome.jp/kegg/pathway.html) and selected DEGs which are confined to the given list of cell proliferation-related genes, identifying 32, 21, and 36 DEGs in VEGF, F1, and Rh1-treated cells, respectively (Fig. 2A,B). Comparison of the cell proliferation-related DEGs showed that none of the DEGs were commonly regulated by VEGF, F1 and Rh1, and that expression of *GNRH1*, *PTK2B*, *LRG1*, and *CSF3* were altered by both F1 and Rh1 treatment, but not by VEGF treatment. We used a similar strategy to analyze the cell migration-related DEGs and identified 15, 6, and 16 DEGS in VEGF, F1, and Rh1-treated cells, respectively (Fig. 2C,D). Three genes (*ZP3*, *FAP*, and *FCER1G)* among the cell migration-related DEGs were similarly regulated by VEGF and ginsenoside (F1 or Rh1). In particular, only the expression of *ZP3*, the gene that encodes zona pellucida sperm-binding protein 3 (ZP3) can be increased with F1, Rh1 and VEGF treatment. Although the molecular mechanism of *ZP3* for cell migration and angiogenesis has been poorly understood, another member of the ZP family, endoglin (ENG), has a crucial role in angiogenesis and the mutation in the ZP domain of ENG is associated with severe human vascular disease^39^, suggesting that *ZP3* is physiologically important to angiogenesis. We also found that expression of *MMP28* and *PTK2B* was reduced in F1 and Rh1-treated cells, while no change was observed upon VEGF treatment. It is noteworthy that *NR4A1* expression, identified as both cell proliferation- and migration-related DEGs, was up-regulated (log_2_ fold-change = 0.80) in VEGF-treated cells, but was significantly down-regulated (log2 fold-change = −1.84) in Rh1-treated cells (Fig. 2A,B). Indeed, we observed that the expression of *NR4A1* was also markedly decreased in two independent replicates of F1-treated cells (Fold change log_2_ = −0.52 in F1-treated cells), although it was not selected as a DEG for cell proliferation and migration in the F1-treated group due to the high p-value (*P*: 0.123805). Altogether, these findings indicate that the molecular mechanism of F1 and Rh1-mediated angiogenesis can be largely different from that of VEGF.

### F1 and Rh1 inhibit VEGF-induced vascular leakage

**Figure 3:**
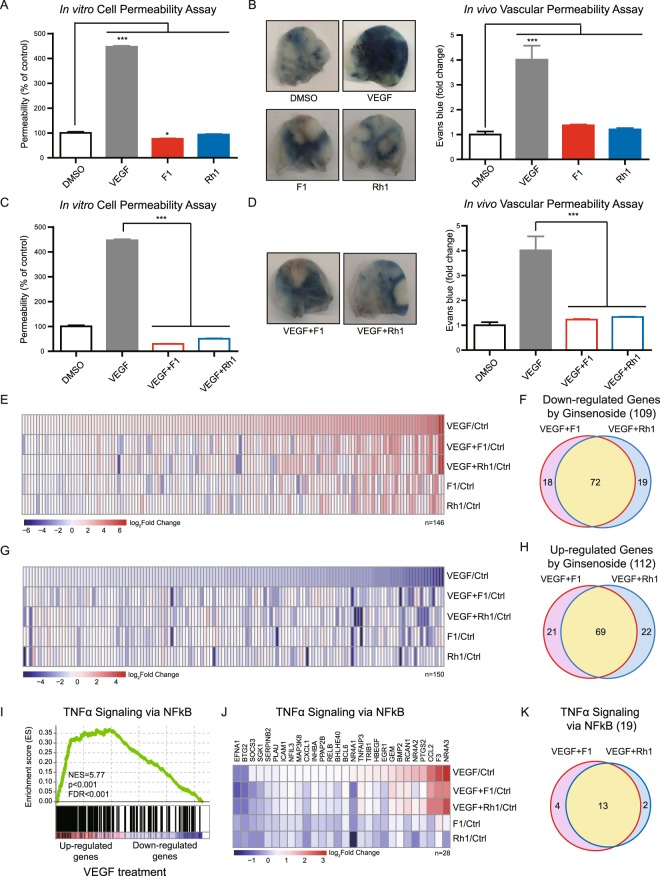
F1 and Rh1 inhibit vascular leakage. (**A**) Effect of F1 and Rh1 on *in vitro* endothelial permeability. HUVECs were treated with VEGF (2.5 nM) or ginsenosides (F1 or Rh1, 10 μM) for an hour and the endothelial permeability was analyzed using the *trans-*well permeability assay with FITC-dextran. Data are presented as mean ± SD (n = 3). (**B**) Effect of F1 and Rh1 on *in vivo* vascular leakage. To visualize plasma leakage, mice ears were treated intradermally with VEGF (250 ng) or ginsenosides (F1 or Rh1, 10 μM in 10 μl of PBS) after intravenous injection of Evans blue dye. Representative images of dye extravasation in mouse ears (left) and its quantification (right). Data are presented as mean ± SD (n = 3). (**C**) Inhibitory effect of F1 and Rh1 on VEGF-induced *in vitro* endothelial permeability. HUVECs were stimulated by VEGF (2.5 nM) for 1 hr and followed by F1 or Rh1 (10 μM) treatment for 1 hr. The endothelial permeability was analyzed by the *trans-*well permeability assay with FITC-dextran. Data are presented as mean ± SD (n = 3). (**D**) Inhibitory effect of F1 and Rh1 on VEGF-induced *in vivo* vascular leakage. To visualize plasma leakage, mice ears were co-treated intradermally with VEGF (250 ng) and ginsenosides (F1 or Rh1, 10 μM in 10 μl of PBS) after intravenous injection of Evans blue dye. Representative images of dye extravasation in mouse ears (left) and its quantification (right). Data are presented as mean ± SD (n = 3). Statistical significance was calculated based on three independent experiments (*P < 0.05; **P < 0.01; ***P < 0.001, P-values between depicted groups). (**E**,**G**) Heatmap representation shows expression changes of VEGF-mediated genes by F1 or Rh1 co-treatment, as compared to DMSO control. Subset of genes that are up-regulated (**E**) and down-regulated (**G**) upon VEGF-treatment. Expression levels of VEGF-mediated genes at indicated condition (log_2_ Fold changes versus DMSO control) were presented by a color range from red (up-regulated) to blue (down-regulated). (**F**,**H**) Venn diagram showing the number of genes with reversed expression levels after VEGF and ginsenoside co-treatment, compared to VEGF treatment alone. Down-regulated genes (**F**) and Up-regulated genes (**H**) by VEGF and ginsenoside co-treatment. (**I**) Enrichment plot of the Gene Set Enrichment Analysis (GSEA) results of VEGF-treated HUVEC. The most significantly enriched gene set, TNFα signaling via NFκB, is shown. NES, Normalized enrichment score; FDR, false discovery rate. (**J**) Heatmap showing gene expression changes at indicated conditions, compared to DMSO control. Among the 123 gene set that belong to ‘TNFα signaling via the NFkB’ pathway, genes whose expression is significantly changed upon VEGF treatment versus DMSO-treated control (Fold change |log_2_| ≥ 0.5, P-value < 0.1) are indicated in Heatmap. (**K**) Among 28 enriched genes that were confined to TNFα signaling via the NFkB pathway, the number of genes, with expression levels that were reversed by VEGF and ginsenoside co-treatment, are indicated in the Venn diagram.

VEGF can induce blood vessel leakage during physiological angiogenesis as well as under pathological conditions such as cancer and age-related macular degeneration^13^. As shown in Fig. 2, the molecular mechanism of F1 and Rh1-mediated angiogenesis seems to be largely different from that of VEGF, therefore, we investigated the role of F1 and Rh1 in vascular leakage. We first assessed the endothelial permeability in response to F1 and Rh1 using the *trans*-well permeability assay in HUVEC or HRMEC monolayers. In contrast to VEGF, F1 and Rh1 showed no significant effect on the trans-endothelial permeability in both HUVECs and HRMECs (Fig. 3A and Supplementary Fig. 5A). We performed a modified Miles vascular permeability assay to further investigate whether F1 and Rh1 induce vascular leakage *in vivo*^34^. Briefly, we intravenously injected Evans blue dye followed by intradermal injection of VEGF, F1, or Rh1 in mice ears and measured the dye extravasation. Consistently, the results showed that unlike VEGF, F1 and Rh1 do not induce vascular leakage (Fig. 3B).

Because acute administration of pro-angiogenic angiopoietin-1 protects against vasculature leakage induced by either VEGF or inflammatory agents^17^, we next examined the effect of F1 and Rh1 on VEGF-induced vascular leakage. We measured the endothelial permeability after treatment with F1 or Rh1, to VEGF-stimulated HUVECs or HRMECs. Surprisingly, VEGF-induced endothelial permeability was significantly suppressed by the additional treatment of F1 or Rh1 (Fig. 3C and Supplementary Fig. 5B). Moreover, the analysis of vascular permeability *in vivo* after the co-injection of F1 or Rh1 with VEGF demonstrated that F1 and Rh1 effectively suppressed the VEGF-induced vascular leakage *in vivo* (Fig. 3D).

To better understand the molecular mechanisms for F1 and Rh1 inhibition of VEGF-induced vascular leakage, we performed RNA-sequencing after treatment with F1 or Rh1 to VEGF-stimulated HUVECs (VEGF + F1 or VEGF + Rh1) and compared the expression levels of genes which are regulated by VEGF (146 up-regulated and 150 down-regulated genes upon VEGF-treatment, Supplementary Table 1). We found of the up-regulated genes upon VEGF-treatment, the expression levels of 109 genes were decreased by at least its 20% upon F1 and Rh1-treatment (Fig. 3E and Supplementary Fig. 6A). Moreover, of the down-regulated genes upon VEGF-treatment, the expressions of 112 genes were increased by at least its 20% upon F1 and Rh1-treatment (Fig. 3G and Supplementary Fig. 6B). Accordingly, treatment of F1 or Rh1 in VEGF-stimulated cells reversed the expression levels of 75% of DEGs in VEGF-treated cells. We also found that 64% of these altered genes overlapped in VEGF + F1 and VEGF + Rh1-treated cells (Fig. 3F–H, and Supplementary Fig. 6A,B), suggesting that F1 and Rh1 considerably change the VEGF-mediated transcriptional responses.

We next conducted gene set enrichment analysis (GSEA) with all genes in VEGF-treated cells compared to DMSO-treated control cells, to further elucidate regulatory mechanisms of F1 and Rh1, and their inhibitory effect on VEGF-induced vascular leakage. GSEA revealed several enriched gene-sets (FDR < 0.06) including angiogenesis, TGF-β signaling, as well as vascular leakage-related responses such as tumor necrosis factor alpha (TNFα) signaling via NFκB, epithelial mesenchymal transition (EMT), inflammatory response, and hypoxia^13,40^ (Supplementary Table 2). In particular, the most significantly enriched gene set was TNFα signaling via NFκB pathway (Fig. 3I), which induces vascular leakage by modulating cell-cell junction integrity^40,41^. Among 28 genes that were included in the “TNFα signaling via NFκB” gene repertoires, the expression of 26 and 2 genes were up- and down-regulated in the VEGF-treated sample, respectively (Fig. 3J). Intriguingly, the expression of 19 genes (68% of 28 genes) in VEGF-treated cells were reduced by at least 20% in VEGF + F1 or VEGF + Rh1-treated cells (Fig. 3K and Supplementary Fig. 6C). Moreover, in both VEGF + F1 and VEGF + Rh1-treated cells, 13 genes (68% of 19 genes, yellow intersection) were commonly regulated (Fig. 3K and Supplementary Figs 6D and 7A), suggesting that these are key targets of both F1 and Rh1 to inhibit VEGF-induced vascular leakage. It has been known that VEGF can induce TNFα expression which play a critical role in VEGF-induced permeability and the major determinant for the inflammatory transcriptional response, NFκB, can be activated by TNFα but not by VEGF^40,42^. To examine the effect of ginsenoside F1 and Rh1 on VEGF-induced signaling, we also investigated the expression of TNFα and the activity of NFκB upon treatment of VEGF and ginsenoside (F1 and Rh1). Surprisingly, VEGF treatment induced the expression of TNFα, which were significantly reversed by subsequent treatment of F1 or Rh1 (Supplementary Fig. 7B,C). However, VEGF treatments or VEGF together with ginsenoside (F1 or Rh1) treatment do not influence the activity of NFκB (Supplementary Fig. 7D). These results indicate that F1 and Rh1 can disturb VEGF-induced TNFα expression and signaling, which is independent of NFκB activity.

We also investigated whether F1 and Rh1 affect VEGF-induced angiogenesis. The gene sets corresponding with cell proliferation- and migration-related DEGs in the VEGF-treated group (Fig. 2) were selected and the expression changes were analyzed under treatment with either VEGF + F1 or VEGF + Rh1. The results indicate that the expressions of 79% and 74% of the DEGs for cell proliferation and migration in VEGF-treated cells were not significantly changed under the condition of VEGF + F1 or VEGF + Rh1 treatment (Supplementary Fig. 8), suggesting that most of the VEGF-induced angiogenic signaling is not disturbed by treatment with F1 or Rh1. Taken together, F1 and Rh1 inhibited VEGF-induced vascular leakage by changing VEGF-mediated transcriptional responses, particularly gene repertoires in “TNFα signaling via NFκB” pathway and TNFα itself, without affecting NFκB activity and VEGF-mediated angiogenesis signaling.

### F1 and Rh1 inhibit vascular leakage via NR4A1 regulation

**Figure 4:**
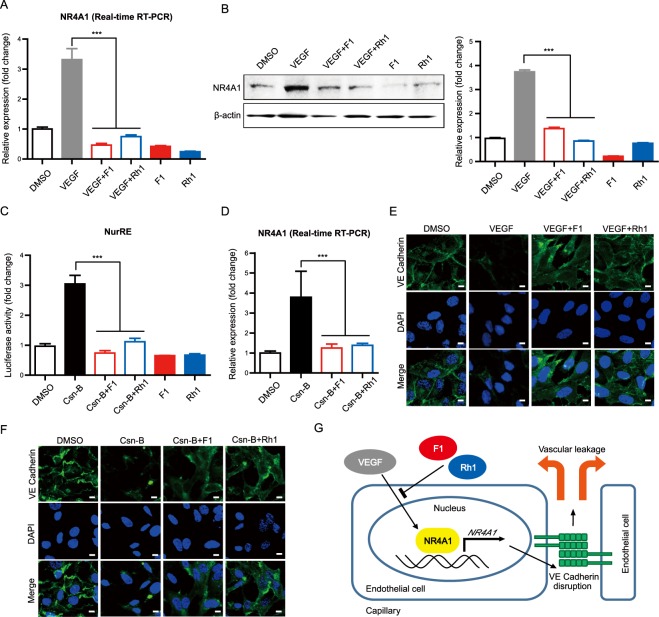
F1 and Rh1 regulate NR4A1 expression and transcriptional activity. (**A**) Effect of F1 and Rh1 on VEGF-induced *NR4A1* mRNA expression in HUVECs. The mRNA expression of *NR4A1* after treatment with F1 or Rh1 in VEGF-stimulated cells was analyzed by Real-time RT PCR. β-actin was used as a reference gene in the analysis. Data are presented as mean ± SD (n = 3). (**B**) Effect of F1 and Rh1 on VEGF-induced NR4A1 protein expression in HUVECs. The protein expressions of NR4A1 after treatment with F1 or Rh1 in VEGF-stimulated cells were assessed with by Western blot analysis. Data are presented as mean ± SD (n = 3). Full-length blots are presented in Supplementary Fig. 9 (**C**) Effect of F1 and Rh1 on NR4A1 transcriptional activity. NurRE-Luc reporter plasmid-transfected HEK293T cells were treated with the indicated condition for 1 hr (0.1 μM of Csn-B and 10 μM of F1 or Rh1). NR4A1 transcriptional activities were analyzed using the dual luciferase reporter gene assay. Data are presented as mean ± SD (n = 3). (**D**) Effect of F1 and Rh1 on Csn-B-induced *NR4A1* mRNA expression in HUVECs. *NR4A1* mRNA expressions after co-treatment of F1 or Rh1 with Csn-B were analyzed by Real-time RT PCR. β-actin was used as a reference gene in the analysis. Data are presented as mean ± SD (n = 3). (**E**) Effect of F1 and Rh1 on the VEGF-induced junctional disruption in HUVECs. The cells were treated with F1 or Rh1 (10 μM, 1 hr) after VEGF stimulation (2.5 nM, 1 hr). VE-cadherin (green) was stained with immunofluorescence and the nucleus (blue) was stained by DAPI. Scale bars, 10 μm. (**F**) Effect of F1 and Rh1 on the Csn-B-induced junctional disruption in HUVECs. The cells were co-treated with 1 μM of Csn-B and 10 μM of F1 or Rh1 for an hour. VE cadherin (green) was stained by immunofluorescence and the nucleus (blue) was stained by DAPI. Scale bars, 10 μm. (**G**) Schematic representation of the inhibitory effect of F1 and Rh1 on VEGF-induced vascular leakage in ECs. Statistical significance was calculated based on three independent experiments (*P < 0.05; **P < 0.01; ***P < 0.001, P-values between depicted groups).

VEGF can cause rapid loss of junctional vascular endothelial (VE)-cadherin and destabilize endothelial cells junctions, thus inducing vascular leakage^43^. NR4A1 (also called TR3, Nur77, NGFI-B, TIS1 and NAK-1), is a member of the nuclear receptor superfamily 4 group A, and is a key mediator of VEGF-induced vascular leakage that increases endothelial nitric-oxide synthase expression and downregulates several endothelial cell junction proteins that maintain vascular homeostasis^22,44,45^. Interestingly, DEG analysis identified NR4A1 as a component related to both cell proliferation- and migration-related DEGs after treatment of VEGF and Rh1 (Fig. 2). In addition, GSEA identified the NR4A family (NR4A1, NR4A2 and NR4A3) as a potential target of both F1 and Rh1 for suppressing VEGF-induced TNFα signaling (Fig. 3). Although we cannot rule out the possibility that ginsenosides can also modulate other factors involved in VEGF-induced hyperpermeability, we chose to first investigate the effect of ginsenosides on NR4A1. Real-time-PCR and western blot clearly demonstrated that the *NR4A1* mRNA and protein levels that are up-regulated by VEGF can be significantly reduced by F1 and Rh1 co-treatment (Fig. 4A,B). Although a physiological ligand for NR4A1 has not been identified, previous studies demonstrated that Cytosporone B1 (Csn-B), which is isolated from *Dothiorella* sp. HTF3, an endophytic fungus, binds to NR4A1 and stimulates transcriptional activity of NR4A1^38^. Because the NR4A1 promoter contains several NR4A1 binding sites, known as the Nur response element (NurRE), Csn-B can also induce gene expression of NR4A1 through positive autoregulation. Therefore, we hypothesized that the steroid-like F1 and Rh1 can directly bind to the NR4A1 nuclear receptor, thus effectively suppressing the transcriptional activity of NR4A1 as well as NR4A1’s expression controlled by autoregulation.

To test this, we first investigated whether F1 and Rh1 can influence the transcriptional activity of NR4A1 after induction with agonist, Csn-B. Human embryonic kidney 293 T (HEK293T) cells were transfected with a luciferase reporter construct that contained a NR4A1 response element (NurRE) where NR4A1 can bind, and the luciferase activities were measured after treatment of Csn-B (0.1 μM) and/or ginsenosides (F1 or Rh1, 10 μM), as indicated. Notably, we found that F1 and Rh1 significantly suppressed Csn-B-stimulated NR4A1 transcriptional activity, suggesting that Csn-B and ginsenoside (F1 and Rh1) can compete with each other for their shared binding pocket in NR4A1 (Fig. 4C). Furthermore, mRNA expression levels of *NR4A1* after treatment of Csn-B (0.1 μM) and ginsenosides (F1 or Rh1, 10 μM) in HUVECs indicate that Csn-B can increase *NR4A1* expression by positive autoregulation, while the co-treatment of Csn-B and ginsenosides (F1 or Rh1) significantly suppressed *NR4A1* expression (Fig. 4D). These results imply that binding of F1 and Rh1 to NR4A1 can effectively suppress not only NR4A1’s transcription activity but also its expression level.

Because VE Cadherin localization and phosphorylation are key parameters for junctional integrity of endothelial cells and vascular leakage, we next investigated the effect of F1 and Rh1 on cellular distribution of junctional VE-cadherin in VEGF-stimulated or Csn-B-stimulated HUVECs. Immunofluorescence staining of VE-cadherin in HUVECs demonstrated that VEGF treatment diminished the junctional VE-cadherin, while VEGF followed by F1 or Rh1 treatment restored VE-cadherin level at the cell-cell junction (Fig. 4E). Similar to VEGF, Csn-B significantly decreased the junctional VE-cadherin and these effects were also blocked by subsequent treatment of F1 or Rh1 (Fig. 4F). These results suggest that NR4A1 mediates VEGF-induced vascular leakage and F1 and Rh1 can effectively inhibit this vascular leakage by suppressing NR4A1’s transcriptional activity as well as decreasing the NR4A1 protein level (Fig. 4G).

## Discussion

Individual ginsenosides have different functional roles in blood vessel and vascular endothelial cells, and exert specific pharmacological effects in pathological conditions^4^. Particularly, Rg1 and Re induce angiogenesis that contributes to wound healing and tissue regeneration, while others, such as Rb1, Rb2 and Rg3 suppress VEGF-induced angiogenesis, leading to tumor growth inhibition^6–10^. Our investigation of 10 individual ginsenosides confirmed the pro-angiogenic effects of Rg1 and Re, and also newly identified that F1 and Rh1 can induce migration and proliferation of endothelial cells to a similar extent as VEGF, Rg1 and Re (Fig. 1). However, *in vitro* trans-well permeability assays and *in vivo* Miles vascular permeability assays clearly demonstrated that F1 and Rh1 did not increase vascular permeability, unlike VEGF (Fig. 3A,B). These results suggest that the molecular mechanism of F1 and Rh1-mediated angiogenesis differs from VEGF-mediated angiogenesis, which is also confirmed by differential expressed genes (DEGs) analysis upon treatment of VEGF, F1 or Rh1. Moreover, the global gene expression profile regulated by F1 and Rh1 as well as their cell proliferation-related and cell migration-related DEGs indicate that a few genes are commonly regulated by F1 and Rh1, suggesting that F1-mediated angiogenesis signaling may also differ from Rh1-mediated angiogenesis.

Vascular permeability can be regulated by VEGF as well as inflammatory mediators, such as TNFα and interleukin-1β (IL-1β)^13,41^. Although the PPD-type ginsenosides, Rb1 and Rk1, inhibit vascular leakage induced by VEGF, lipopolysaccharide (LPS), thrombin, or histamine^20,21^, no pro-angiogenic ginsenoside has been reported to reduce endothelial permeability. Since Rg1 is known to induce VEGF expression through glucocorticoid receptor activation^46^, pro-angiogenic Rg1 would increase vascular permeability. Notably, our data revealed that pro-angiogenic F1 and Rh1 prevent undesirable vascular leakage induced by VEGF both *in vitro* and *in vivo* (Fig. 3C,D). Moreover, the expression of 75% up- or down-regulated genes upon VEGF treatment were reversed by co-treatment of VEGF with F1 or Rh1 and 64% of these altered genes overlapped in VEGF + F1 and VEGF + Rh1-treated cells (Fig. 3E–H), suggesting that F1 and Rh1 considerably change the VEGF-mediated transcriptional responses with a similar signaling pathway. Furthermore, our comparative transcriptome analysis identified that the effects of F1 and Rh1 on endothelial cell barrier function and restoration are mainly attributed to the changes in VEGF-mediated TNFα signaling via NFκB, particularly to the suppressed transcriptional activity of the orphan nuclear receptor, NR4A1 and its decreased gene expression by F1 and Rh1 treatment. More interestingly, F1 and Rh1 significantly disturbed the expression of TNFα itself which are induced by VEGF treatment. Consistent with our results, recent studies have shown that F1 and Rh1 can ameliorate myocardial injury by inhibiting TNF-α and IL-1β expression^47^. Because genes induced by VEGF or IL-1β largely overlap (~60%) and both inflammation and angiogenesis are interconnected with each other^48^, F1 and Rh1 can be a dual-modulator of vascular permeability and inflammation.

NR4A1, an orphan nuclear receptor, is strongly and immediately up-regulated by VEGF, and its transcriptional activity is critical not only for inducing angiogenesis but also for down-regulating endothelial cell junctional proteins such as VE-cadherin, claudin and occludin, thus increasing vascular leakage^45,49^. Our data indicate that the transcriptional activation of NR4A1 by the NR4A1-specific agonist, Csn-B, reduces the junctional VE-cadherin level to a similar extent as VEGF (Fig. 4E,F), confirming that NR4A1 is a key mediator for vascular permeability. Surprisingly, F1 and Rh1 can significantly restore VEGF- or Csn-B-induced vascular leakage, suggesting that F1 and Rh1 specifically inhibit NR4A1, which can be an effective therapeutic candidate for blocking vascular leakage in ischemic disease and cancer. Since NR4A1 is also involved in numerous other physiological roles such as metabolic processes, inflammation, steroidogenesis, and brain function, it has been considered a multi-functional drug target by manipulating NR4A’s expression levels, transactivation activity, subcellular localization, or interactions with other proteins. For example, the NR4A1-specific agonist, Csn-B, induces apoptosis by either target gene expression or by facilitating the translocation of NR4A1 to the mitochondria^38^. The NR4A1-targeting compound, ethyl 2-[2,3,4-trimethoxy-6-(1-octanoyl)phenyl] acetate (TMPA) reduces blood glucose by disrupting NR4A1-LKB1 interaction and activating AMPKα^50^, and 1-(3,4,5-trihydroxyphenyl)nonan-1-one (THPN) induces autophagic cell death of melanoma through the mitochondrial signaling pathway^51^. More interestingly, n-pentyl 2-[3,5-dihydroxy-2-(1-nonanoyl)-phenyl] acetate (PDNPA) can attenuate LPS-induced inflammation by blocking the NR4A1-p38α interaction^52^. Recent structural studies on NR4A1 in complex with these specific targeting compounds (TMPA, THPN and PDNPA) have shown that they bind to the surface of ligand binding domain (LBD) of NR4A1 rather than in the ligand-binding pockets deep in the protein interior, thus modulating NR4A1’s subcellular localization, or interactions with other proteins^50–52^. Although we cannot rule out the possibility in which the inhibitory effect of F1 and Rh1 on vascular leakage can be attributed by the modulation of interactions between NR4A1 and other proteins like specific targeting compounds (TMPA, THPN and PDNPA), our data from the NurRE reporter assay indicate that these effects of F1 and Rh1 are primarily caused by direct regulation of NR4A1’s transcriptional activity (Fig. 4C). Therefore, it is not yet clear whether F1 and Rh1 similarly bind to NR4A1 as specific targeting compounds (TMPA, THPN and PDNPA) or bind to the interior pocket of NR4A1, similar to steroid-like ligand binding to target nuclear receptors for their transcriptional regulation. Further in-depth structural study will be required to fully understand the inhibitory function of F1 and Rh1 on NR4A1-mediated vascular leakage as well as the molecular mechanism for competition between Csn-B and ginsenoside (F1 and Rh1).

Among ginsenoside variants, the major ginsenosides, PPD-type (Rb1, Rb2, Rb3, Rc and Rd) and PPT-type (Re and Rg1), comprise more than 80% of total ginsenoside content. As shown the novel effects of F1 and Rh1 on angiogenesis and vascular permeability in our study, increasing evidences for medical effects of each minor ginsenosides have been accumulating. However, minor ginsenosides are rarely present in natural ginseng. Although numerous methods, including physical (heat treatment), chemical (acid or base treatment) and biological (microorganisms or enzymes) transformation have been explored to convert major ginsenoside into more pharmacologically active minor ginsenosides^27^, the large-scale production of individual minor ginsenosides by traditional methods is not practical due to its complexity and high cost, which also further limits pharmacological research and new drug development. In the near future, synthetic biology strategies that introduce the key enzymes involved in ginsenoside biosynthesis into yeast or bacteria could provide a key manufacturing approach to produce individual minor ginsenosides such as F1 and Rh1 for therapeutic development^53,54^.

In conclusion, our investigation of 10 different ginsenoside variants reveal that PPT-type F1 and Rh1 are unique ginsenoside variants that exert both pro-angiogenic and anti-vascular leakage effects. Moreover, we identify an orphan nuclear receptor NR4A1, which is a key mediator of VEGF-induced vascular leakage, as a binding target of F1 and Rh1. Ginsenosides F1 and Rh1 alone or in conjunction with other therapeutics can be a promising remedy for vessel normalization in ischemic disease and cancer.

## Supplementary information

Supplementary Information
