# Supplementary material for: Pro-angiogenic Ginsenosides F1 and Rh1 Inhibit Vascular Leakage by Modulating NR4A1
Source: Sci Rep. 2019 Mar 14;9:4502. doi: 10.1038/s41598-019-41115-2 (PMC6418182; doi:10.1038/s41598-019-41115-2)
Supplement: Supplementary file 1 — Supplementary Information [file 41598_2019_41115_MOESM1_ESM.docx]

**Pro-angiogenic Ginsenosides F1 and Rh1 Inhibit Vascular Leakage by Modulating NR4A1**

**Ji In Kang^1#^, Yoonjung Choi^2#^, Chang-Hau Cui^3^, Daeyoup Lee^2*^, Sun Chang Kim^2,3*^, Ho Min Kim^1,4,5*^**

^1^Biomedical Science and Engineering Interdisciplinary Program, Korea Advanced Institute of Science and Technology (KAIST), 291 Daehak-ro, Yuseong-gu, Daejeon 34141, Korea

^2^Department of Biological Sciences, Korea Advanced Institute of Science and Technology (KAIST), 291 Daehak-ro, Yuseong-gu, Daejeon 34141, Korea

^3^Intelligent Synthetic Biology Center, 291 Daehak-ro, Yuseong-gu, Daejeon 34141, Korea

^4^Center for Biomolecular & Cellular Structure, Institute for Basic Science (IBS), Daejeon 34126, Korea

^5^Graduate School of Medical Science & Engineering, Korea Advanced Institute of Science and Technology (KAIST), 291 Daehak-ro, Yuseong-gu, Daejeon 34141, Korea

^#^ These authors contributed equally to this study

*Correspondence

Ho Min Kim, hm_kim@kaist.ac.kr

Sun Chang Kim, sunckim@kaist.ac.kr

Daeyoup Lee, dylee@kaist.ac.kr

**Key words:** ginsenoside F1; ginsenoside Rh1; angiogenesis; vascular leakage; nuclear receptor subfamily 4 group A member 1; vascular endothelial-cadherin

**Supplementary Figures**


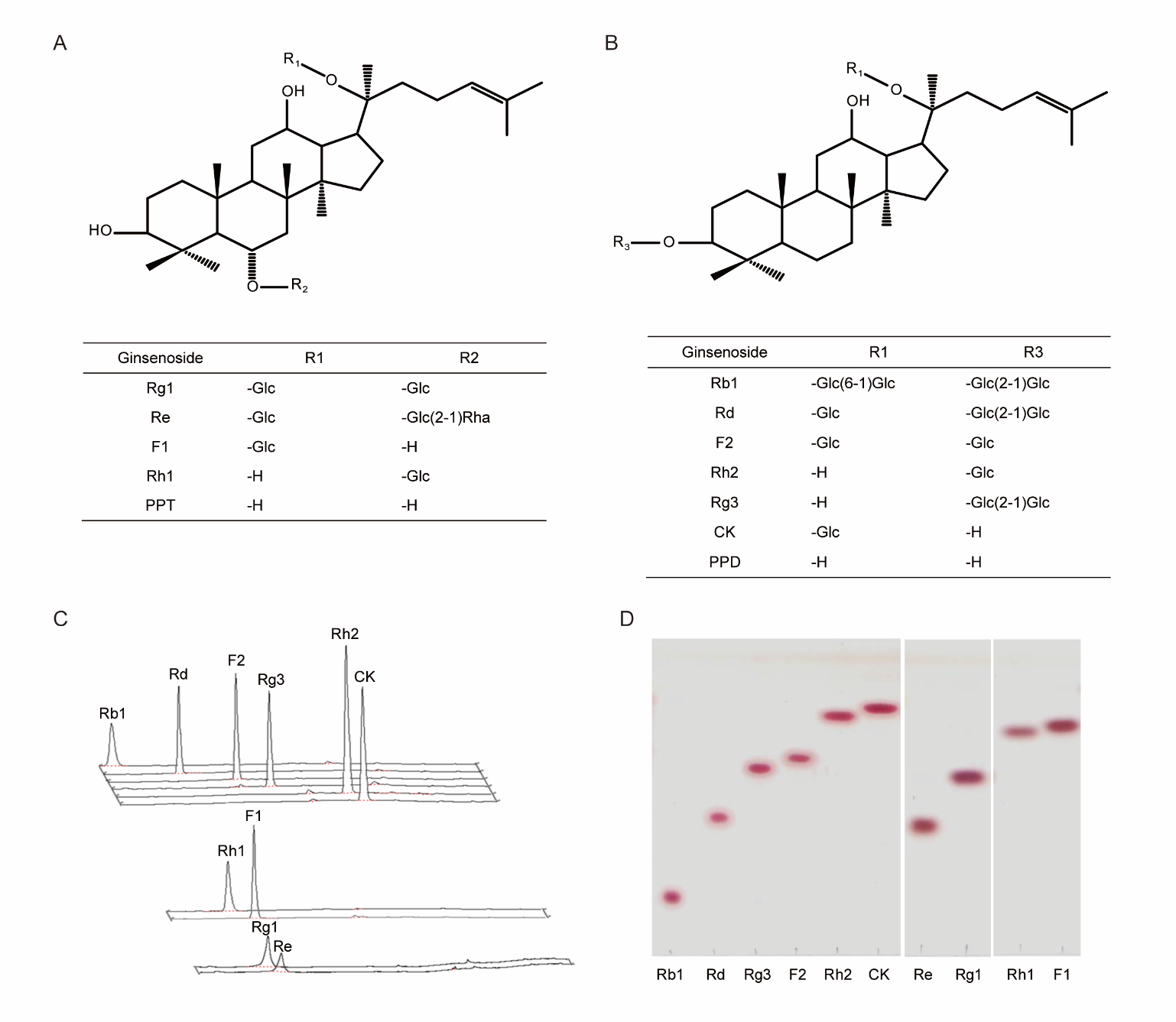


**Supplementary Figure 1. Preparation of ginsenosides. (A)** Chemical structures of protopanaxatriol (PPT) ginsenosides. **(B)** Chemical structures of protopanaxadiol (PPD) ginsenosides. Glc: β-D-glucose; Rha: α-L-rhamnose. **(C and D)** The highly purified (>95%) major ginsenoside (Rg1, Re, Rb1, Rd) and minor ginsenoside (F1, F2, Rh1, Rh2, Rg3 and CK) were produced as described in the methods section. The purity of the ginsenoside variants was evaluated by **(C)** high-performance liquid chromatography (HPLC) and **(D)** thin-layer chromatography (TLC) analysis.


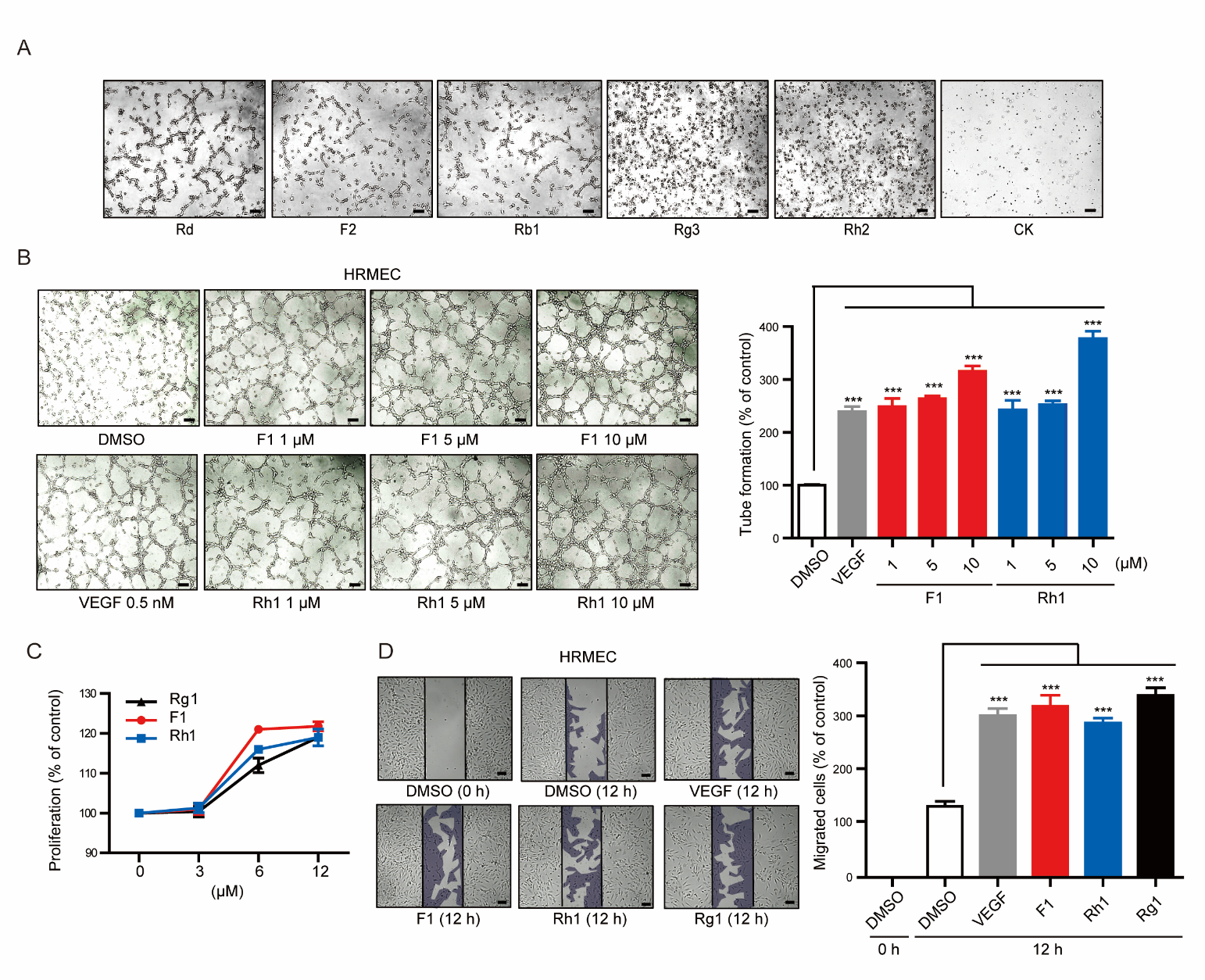


**Supplementary Figure 2. F1 and Rh1 promote tube formation, proliferation, and migration of HUVECs and HRMECs. (A)** Representative images for the tube formation assay are shown in HUVECs with the treatment of PPD-type ginsenosides (25 μM) for 4 hr. Scale bars, 100 μm. **(B)** Dose-dependent effects of F1 and Rh1 on tube formation in HRMECs. Tube formation was measured after the treatment with DMSO as control, VEGF (0.5 nM), or indicated ginsenosides (25 μM) for 4 hr. Representative images (left) and the quantification (right) of the tube formation assay are shown. Scale bars, 100 μm. Data are presented as mean ±SD (n =3). **(C)** Effects of F1 and Rh1 on cell proliferation in HRMECs. HRMEC were treated with the indicated concentrations of F1, Rh1, or Rg1 for 24 hr and cell proliferation was measured by a WST-1 assay. **(D)** Effects of F1 and Rh1 on cell migration in HRMECs. The cell migration assay was conducted in HRMECs that were treated with VEGF (2.5 nM) or indicated ginsenosides (25 μM) for 12 hr. Representative cell migration images are shown and the wound-healing areas are indicated in blue (left). Scale bars, 100 μm. The migration areas were measured by ImageJ software and the quantitative data are presented as mean ±SD (n =3) (right). Statistical significance was calculated based on three independent experiments (*P < 0.05; **P < 0.01; ***P < 0.001, P-values between depicted groups).

**
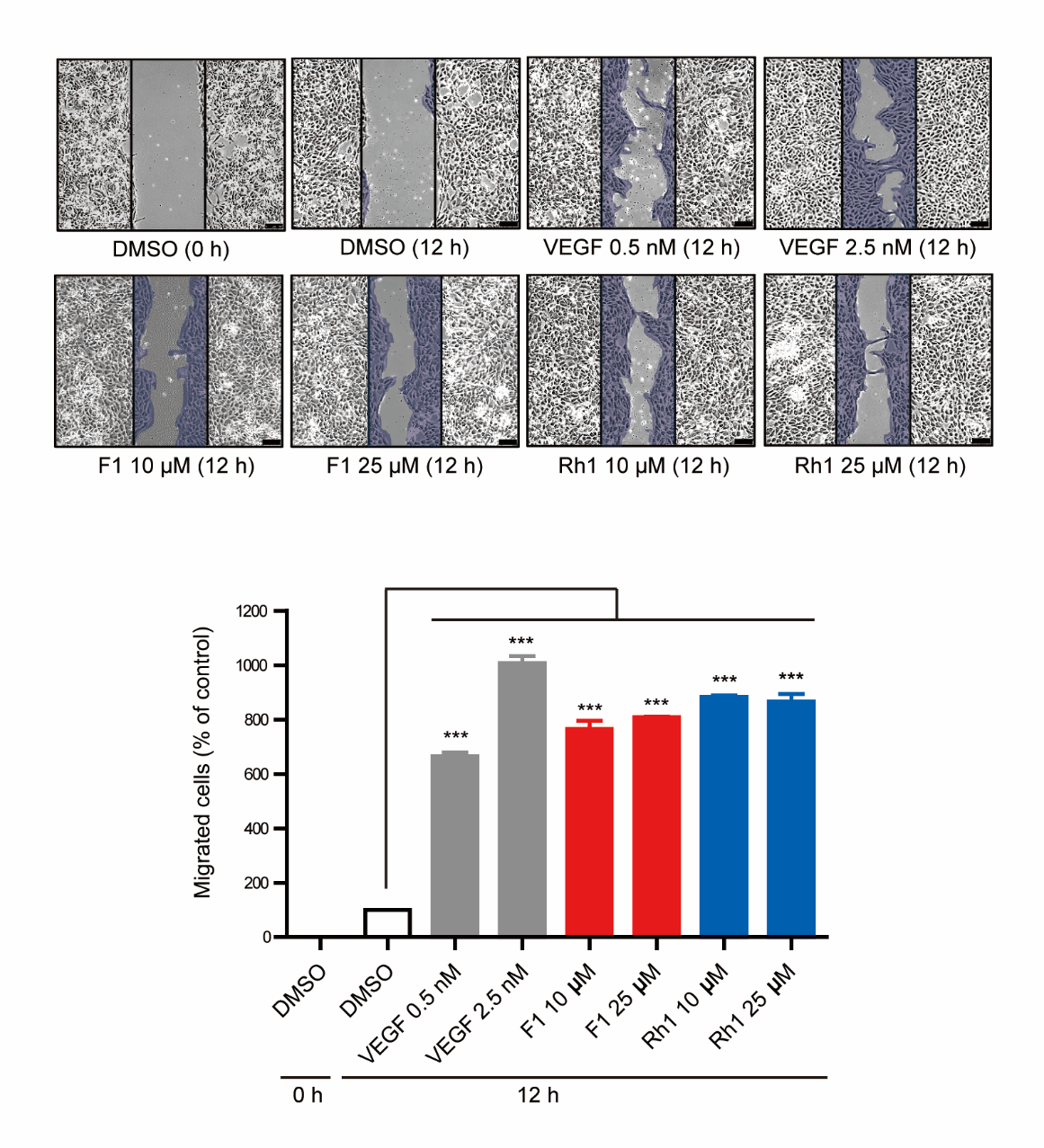
**

**Supplementary Figure 3. Effects of F1 and Rh1 on cell migration in HUVECs.** The cell migration assay was conducted in HUVECs treated with VEGF (0.5nM or 2.5 nM) and indicated ginsenosides (10 μM or 25 μM) for 12hr. Representative cell migration images are shown and the wound-healing areas are indicated in blue (top). Scale bars, 100 μm. The migration areas were measured by ImageJ software and the quantitative data are presented as mean ±SD (n =3) (bottom). Statistical significance was calculated based on three independent experiments (*P < 0.05; **P < 0.01; ***P < 0.001, P-values between depicted groups).

**
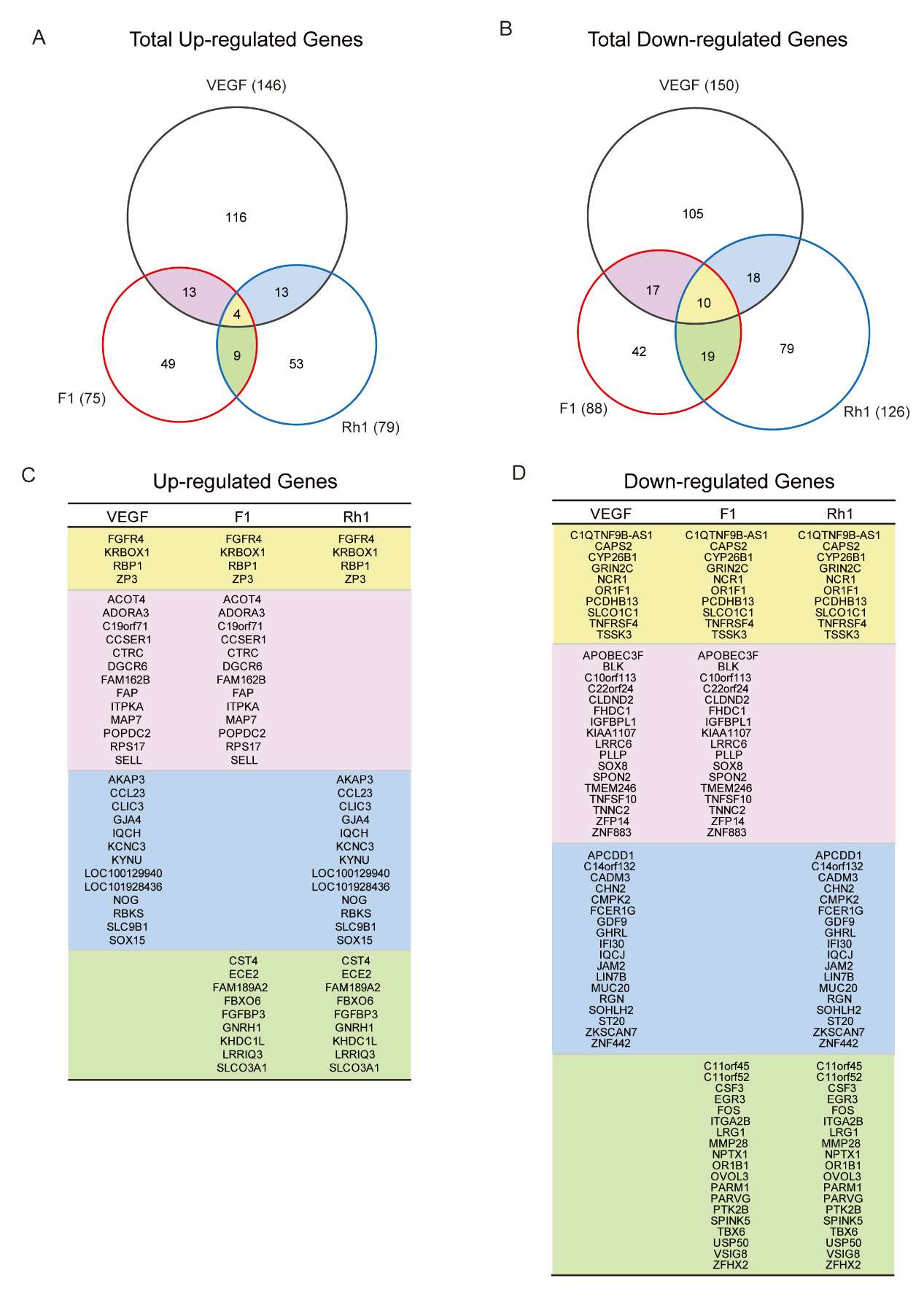
**

**Supplementary Figure 4. Global profiles of gene expression regulated by ginsenosides and VEGF. (A and B)** Venn diagrams showing the overlapping subsets of differentially expressed genes (DEGs) for up-regulated **(A)** and down-regulated genes **(B)** in VEGF, F1 and Rh1 treatment versus the DMSO-treated control sample (Fold change |log2| ≥ 0.5, P-value < 0.1). **(C and D)** Tables showing the list of overlapping DEGs, up-regulated **(C)** and down-regulated genes **(D)**, with the same color as the Venn diagram intersection in **A** and **B**.

**
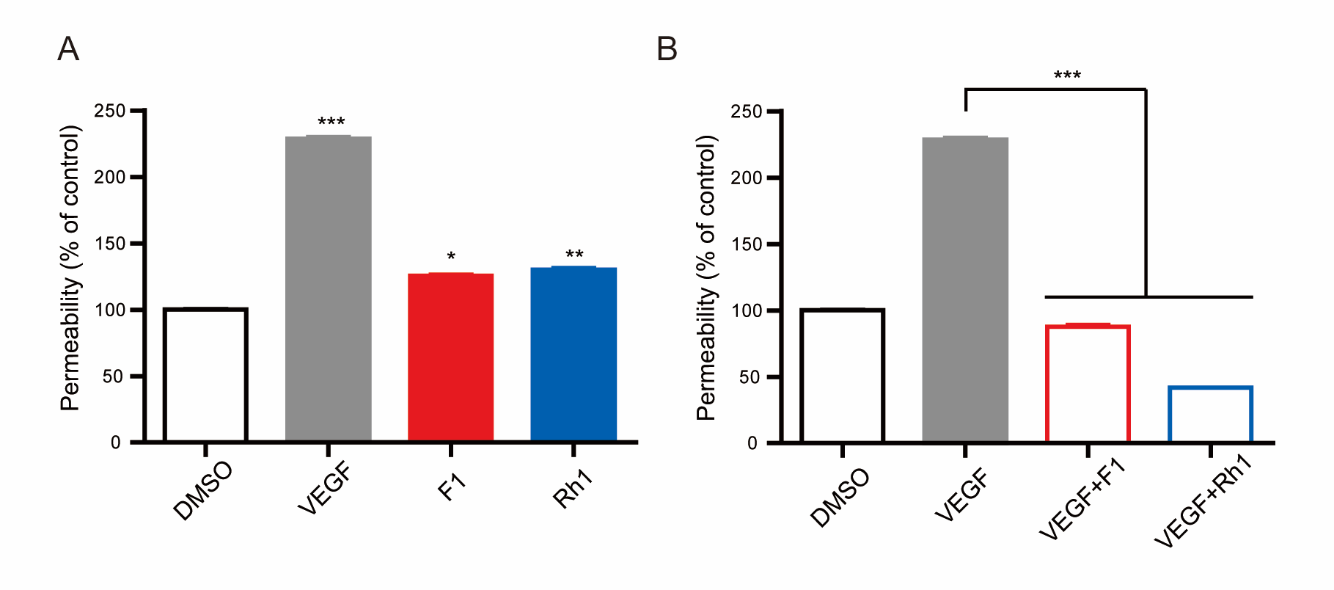
**

**Supplementary Figure 5. F1 and Rh1 inhibit endothelial permeability in HRMECs.** **(A)** Effect of F1 and Rh1 on in vitro endothelial permeability. HRMECs were treated with VEGF (2.5 nM) or ginsenosides (F1 or Rh1, 10 μM) for an hour and the endothelial permeability was determined using the trans-well permeability assay with FITC-dextran. Data are presented as the mean ±SD (n =3). **(B)** The inhibitory effect of F1 and Rh1 on VEGF-induced in vitro endothelial permeability. HRMECs were stimulated by VEGF (2.5 nM) for 1 hr followed by F1 or Rh1 (10 μM) treatment for 1 hr. The endothelial permeability was analyzed by the trans-well permeability assay with FITC-dextran. Data are presented as the mean ±SD (n =3). Statistical significance was calculated based on three independent experiments (*P < 0.05; **P < 0.01; ***P < 0.001, P-values between depicted groups).

**
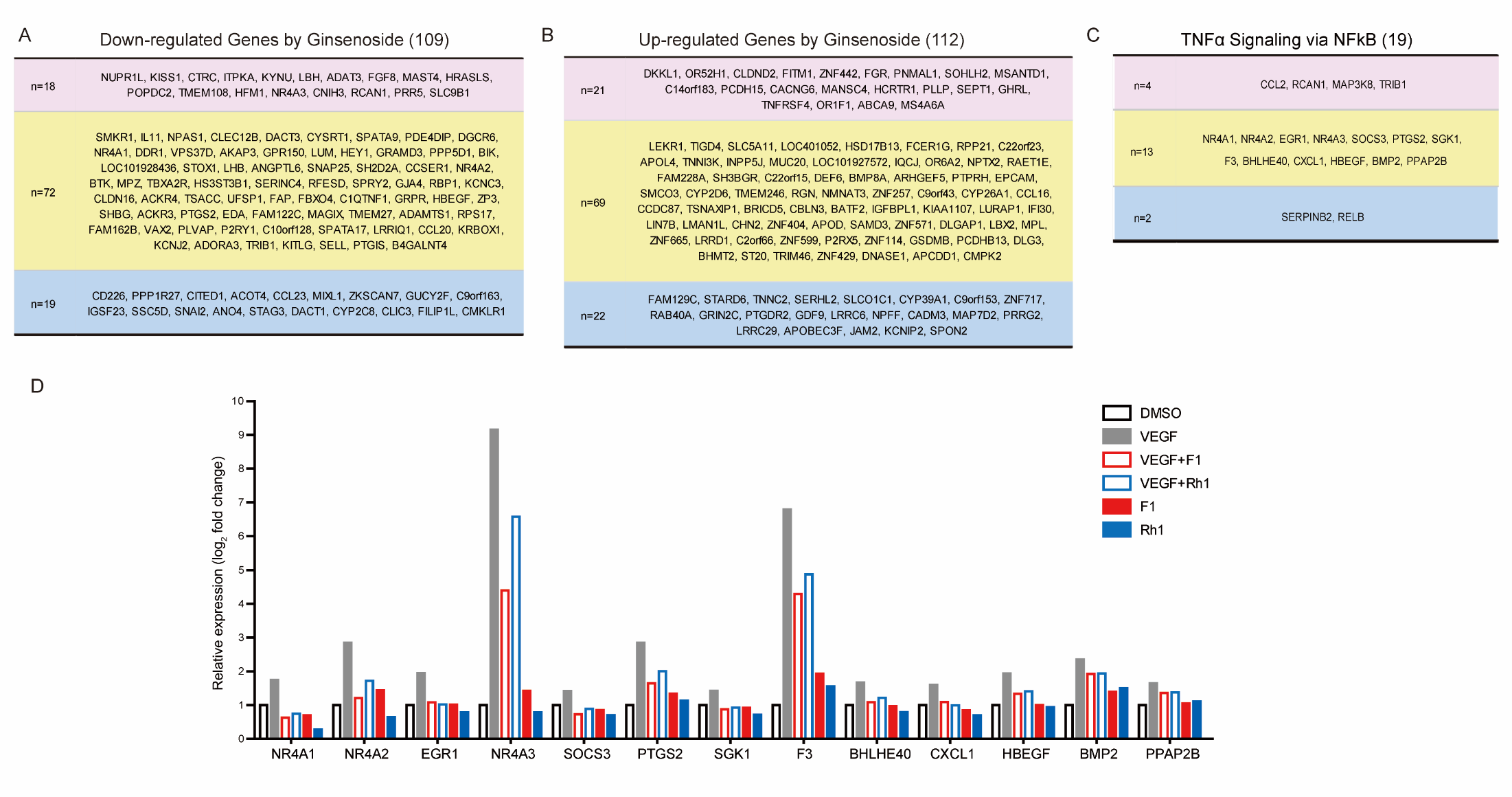
**

**Supplementary Figure 6. F1 and Rh1 regulate VEGF-induced TNFα signaling.** **(A-C)** Entire gene lists presented in the Venn diagram of **Figures 3F (A), 3H (B) and 3K (C)**. The color of the table matches the color of the Venn diagram region in Figures **3F, 3H** and **3K**. **(D)** The relative expressions of 13 genes which corresponded to the Venn diagram intersection of Figure **3K**.

**
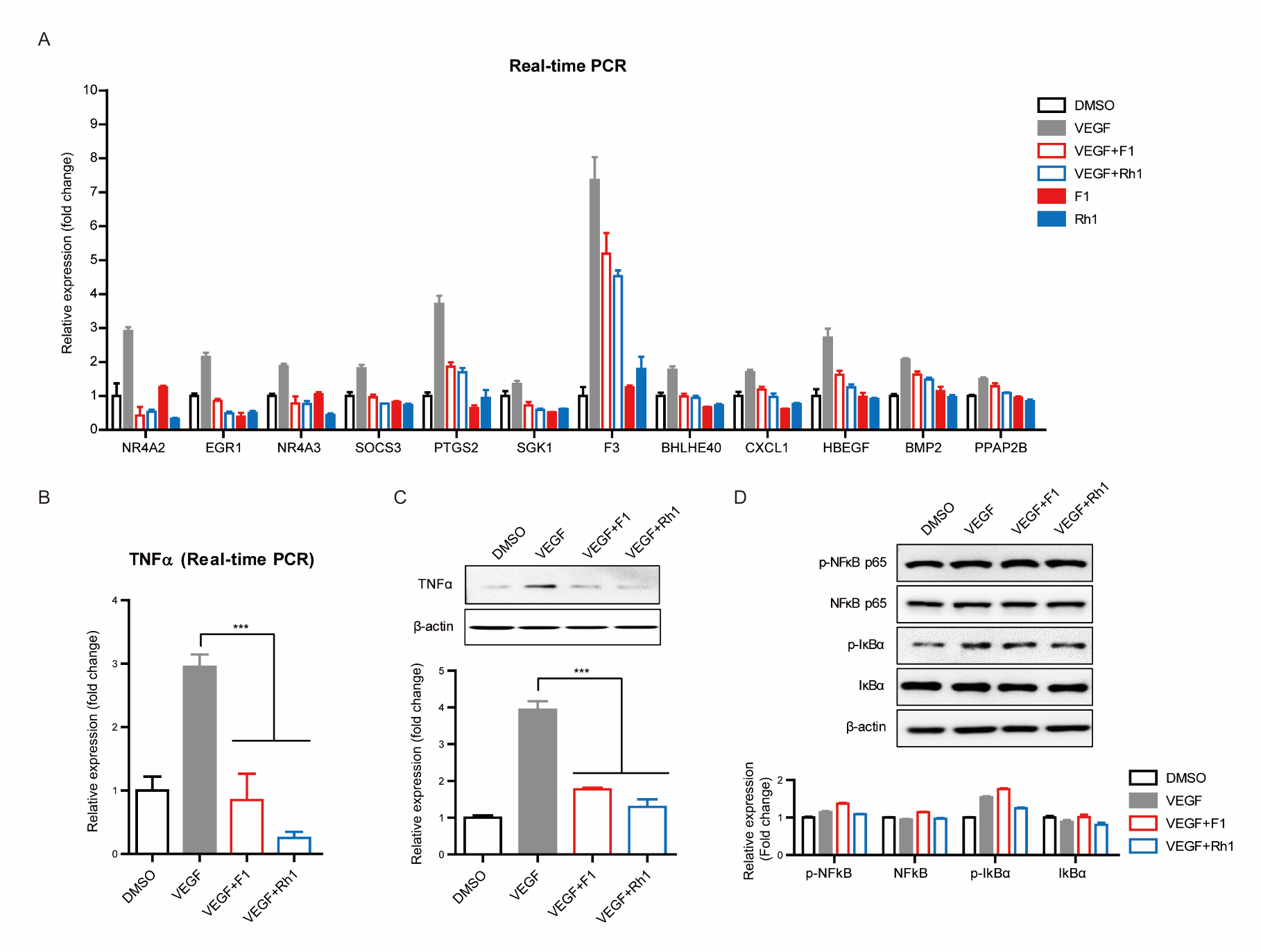
**

**Supplementary Figure 7. F1 and Rh1 regulate VEGF-induced TNFα signaling and TNFα expression, but not NFkB activity.** **(A)** The relative expressions of TNFα signaling-related genes which corresponded to the Venn diagram intersection of Figure **3K** were analyzed by real-time PCR at indicated condition. Data are presented as mean ±SD (n =3). **(B-C)** The amount of mRNA **(B)** and protein **(C)** of TNFα were analyzed by real-time PCR and Western blot analysis at indicated condition. Data are presented as mean ±SD (n =3). **(D)** NFκB activation was analyzed by Western blot analysis at indicated condition. Data are presented as mean ±SD (n =3). Statistical significance was calculated based on three independent experiments (**P* < 0.05; ***P* < 0.01; ****P* < 0.001, P-values between depicted groups). Full-length blots are presented in Supplementary Fig. 9.

**
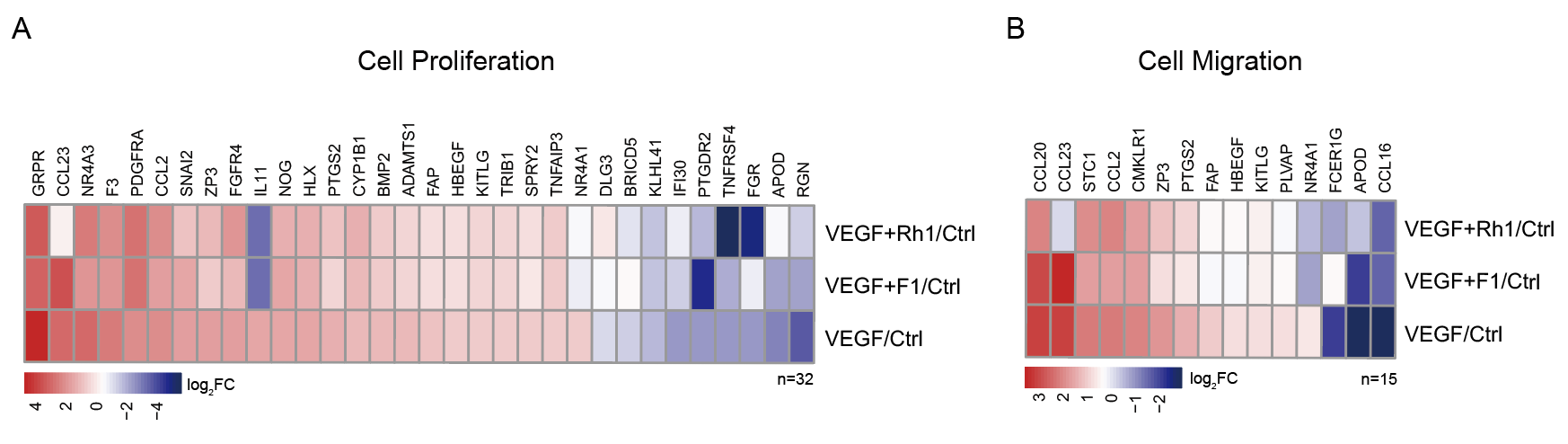
**

**Supplementary Figure 8. Effects of F1 and Rh1 on VEGF-induced angiogenic signaling.** Heatmap representation showing gene expression changes of VEGF-induced DEGs for cell proliferation **(A)** and for cell migration-related genes **(B)** in the VEGF+F1, and VEGF+Rh1 treatment groups versus the DMSO control group. Expression levels are shown as relative values (log2) normalized to the DMSO-treated control. Red, up-regulated; blue, down-regulated.


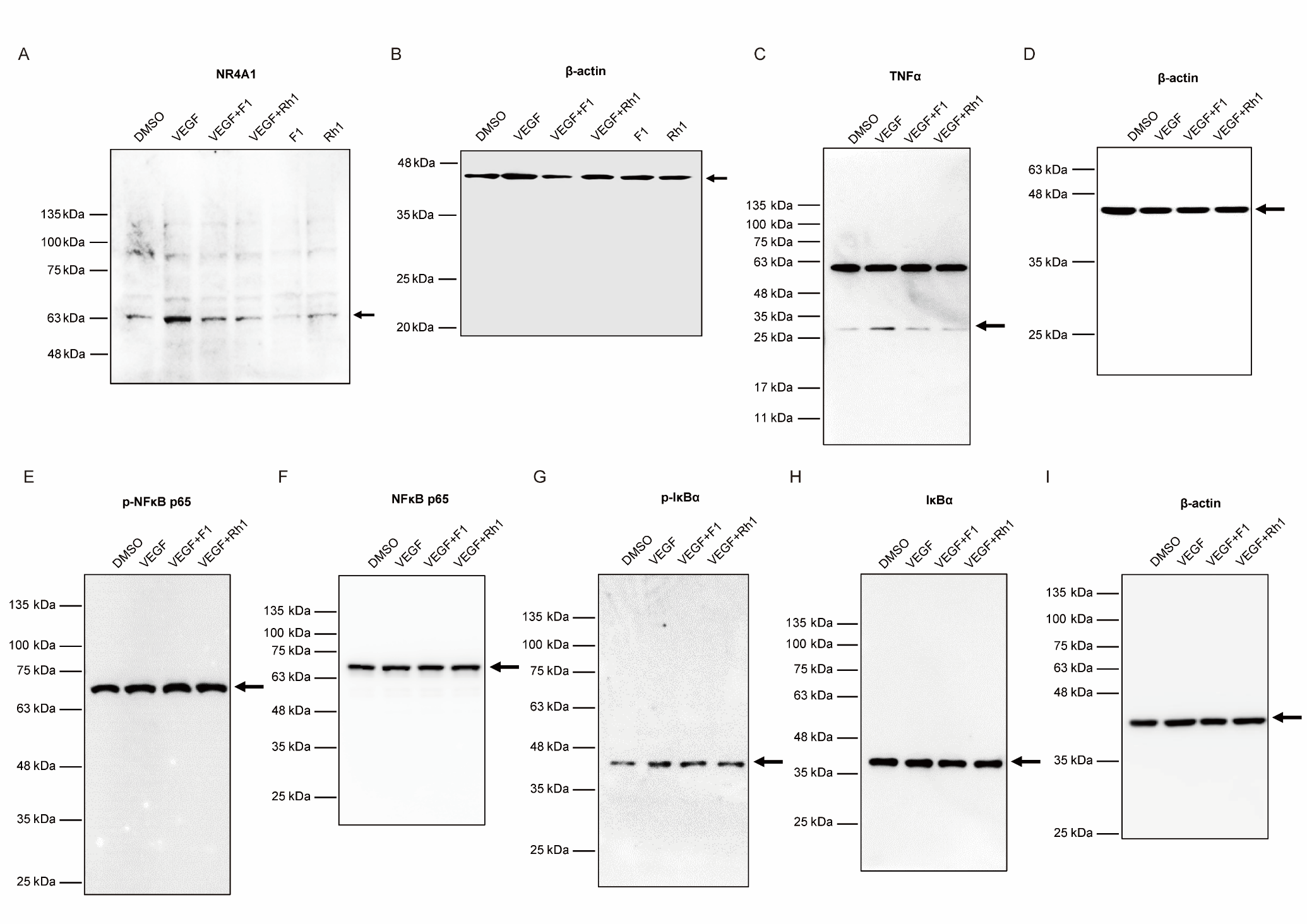


**Supplementary Figure 9. Full-length blot of Figure 4B and Supplementary Figure 7C-D.**

**Supplementary Tables**

**Supplementary Table 1.** Differentially expressed genes in VEGF, F1, or Rh1-treated HUVECs compared to DMSO-treated control cells, Fold change |log_2_| ≥ 0.5, P-value < 0.1

| **A.** Up-regulated genes | |
| --- | --- |
| VEGF | ACKR3, ACKR4, ACOT4, ADAMTS1, ADAT3, ADORA3, AKAP3, ALS2CR11, ANGPTL6, ANO4, ARHGDIG, ARTN, ASIC2, B4GALNT4, BAALC, BIK, BMP2, BTK, C10orf128, C19orf71, C1QTNF1, C2CD4B, C9orf163, CA8, CASP1, CCDC103, CCL2, CCL20, CCL23, CCSER1, CD226, CEMIP, CITED1, CLDN14, CLDN16, CLEC12B, CLIC3, CMKLR1, CNIH3, CST6, CTRC, CXCL3, CYP1B1, CYP2C8, CYSRT1, DACT1, DACT3, DDR1, DGCR6, DKK2, EDA, F3, FAM122C, FAM162B, FAP, FBXO4, FGF8, FGFR4, FILIP1L, GJA4, GPR150, GRAMD3, GRPR, GUCY2F, HBEGF, HEY1, HFM1, HLX, HRASLS, HS3ST3B1, IGSF23, IL11, IQCH, ITPKA, KCNC3, KCNJ2, KIAA0513, KISS1, KITLG, KRBOX1, KYNU, LBH, LHB, LOC100129940, LOC101928436, LRRIQ1, LUM, MAGIX, MAP7, MAST4, MIXL1, MPZ, NEDD9, NOG, NPAS1, NPB, NR4A1, NR4A2, NR4A3, NUPR1L, P2RY1, PDE4DIP, PLVAP, POPDC2, PPP1R27, PPP5D1, PRR5, PTGIS, PTGS2, RBKS, RBP1, RCAN1, RFESD, RPS17, SELE, SELL, SERINC4, SH2D2A, SHBG, SLC30A3, SLC9B1, SMKR1, SNAI2, SNAP25, SOX15, SPATA17, SPATA9, SPRY2, SSC5D, STAG3, STC1, STOX1, TBXA2R, TFR2, TMEM108, TMEM27, TNFAIP3, TRIB1, TSACC, TSPAN33, UFSP1, UPB1, VAX2, VPS37D, ZKSCAN7, ZP3 |
| F1 | ABLIM2, ACOT4, ADORA3, ARL17A, ATP6AP1L, AZIN2, BCAS4, BCL2L15, C12orf79, C19orf71, CATSPER1, CCSER1, CMTM2, COX8C, CPLX1, CRYAB, CST4, CTRC, DGCR6, DLGAP1, ECE2, ERICH6, FAAH, FAM162B, FAM189A2, FAP, FBLN2, FBXO6, FGFBP3, FGFR4, FNDC7, FOXD4L1, GADD45G, GNMT, GNRH1, GPBAR1, HK3, HOXC5, IL37, ITIH4, ITPKA, KHDC1L, KRBOX1, LINGO1, LMO1, LOC100287036, LRRIQ3, MAP7, MESP1, NLRP12, OR52E6, PATE2, PKIB, POPDC2, PPP1R14A, PRG3, RASGRP2, RBP1, RFX8, RPS17, RUSC1-AS1, SCNN1D, SELL, SLC22A1, SLCO3A1, SRRM3, TEX12, TFF3, TMCO2, TMEM210, TMEM30B, TOMM20L, VASN, VSIG10L, ZP3 |
| Rh1 | ACSM3, AKAP14, AKAP3, ANKRD35, APLP1, ARL13A, BIRC3, BTBD8, C2orf81, CAMK4, CAPN3, CBY3, CCDC151, CCDC181, CCL23, CFAP57, CGREF1, CLIC3, CST4, ECE2, ETV7, EXOC3L2, FAM189A2, FAM69A, FBXO6, FGFBP3, FGFR4, GJA4, GNRH1, GOLGA8N, GS1-259H13.2, HAS3, IL17C, IQCH, ITGA11, KCNC3, KHDC1L, KLF15, KRBOX1, KYNU, LGALS3BP, LOC100129940, LOC101928436, LRRIQ3, NAP1L2, NMNAT2, NOG, PAK6, PCDHA1, PCDHA5, PPM1H, RAB3A, RBKS, RBP1, RGS7, RHCE, RNASE6, RSPH1, RTN4RL2, SCGB2B2, SEMA3C, SEMA7A, SLC9B1, SLCO3A1, SOX15, SSTR5, SULT1E1, TCTE3, TFF1, TIAM1, TSGA10, TTC39C, TXK, VAV3, WFDC3, ZNF114, ZNF19, ZNF474, ZP3 |
|  |  |
| **B.** Down-regulated genes | |
| VEGF | ABCA9, ABCG4, ANO8, APCDD1, APOBEC3F, APOD, APOL4, ARHGEF5, BAIAP3, BATF2, BHMT2, BLK, BMP8A, BRICD5, C10orf113, C14orf132, C14orf183, C1QTNF9B-AS1, C20orf195, C22orf15, C22orf23, C22orf24, C2orf66, C9orf153, C9orf43, CACNG6, CADM3, CAPS2, CBLN3, CCDC87, CCL16, CHN2, CLDND2, CMPK2, CRYBB3, CYP26A1, CYP26B1, CYP2D6, CYP39A1, DEF6, DKKL1, DLG3, DLGAP1, DNASE1, DNM1, EPCAM, FAM129C, FAM228A, FCER1G, FGR, FHDC1, FITM1, FMO5, GDF9, GHRL, GRIN2C, GSDMB, HCRTR1, HSD17B13, IFI30, IGFBPL1, INPP5J, IQCJ, JAM2, KCNIP2, KCNS1, KIAA1107, KLHL41, LBX2, LEKR1, LIN7B, LMAN1L, LOC101927572, LOC401052, LRRC29, LRRC6, LRRC75A, LRRD1, LURAP1, LYNX1, MANSC4, MAP7D2, MDH1B, MPL, MS4A6A, MSANTD1, MUC20, NCR1, NMNAT3, NPFF, NPTX2, NYAP1, OR1F1, OR52H1, OR6A2, P2RX5, PCDH15, PCDHB13, PCDHGA8, PIK3IP1, PLLP, PLXNA4, PNMAL1, PRRG2, PTGDR2, PTPRH, RAB40A, RAET1E, RASSF5, RDM1, RGN, RPP21, SAMD3, SEPT1, SERHL2, SH3BGR, SLC5A11, SLCO1C1, SMCO3, SOHLH2, SOX8, SPON2, ST20, STARD6, TCEA3, TIGD4, TMEM246, TNFRSF4, TNFSF10, TNNC2, TNNI3K, TRIM46, TSNAXIP1, TSSK3, TTC23L, ZFP14, ZKSCAN7, ZNF114, ZNF221, ZNF257, ZNF404, ZNF429, ZNF442, ZNF571, ZNF599, ZNF665, ZNF717, ZNF846, ZNF883, ZSCAN31 |
| F1 | ABAT, ADIG, AKR1B10, APOBEC3F, ASPRV1, BEGAIN, BEX5, BLK, C10orf113, C11orf45, C11orf52, C17orf100, C1orf53, C1QTNF9B-AS1, C22orf24, CAPS2, CLDND2, CLEC12B, CREB3L1, CSF3, CYP26B1, DDX43, EGFLAM, EGR3, FHDC1, FOS, GDPGP1, GPR25, GRIN2C, HGF, IGFBPL1, ITGA2B, ITM2A, KALRN, KIAA1107, KLHDC9, LRG1, LRRC6, MEIG1, MFSD7, MMP28, MYL4, NCR1, NPTX1, NRG3, OR1B1, OR1F1, OVOL3, PARM1, PARVG, PCDHB13, PCDHGC5, PFKFB1, PLLP, PPP1R3F, PTK2B, RARRES1, RASL10B, RUNDC3B, SCN10A, SEC16B, SLC22A17, SLC45A2, SLCO1C1, SMIM17, SMPDL3B, SOX8, SPATA32, SPINK5, SPON2, SPTLC3, SYTL2, TBX6, TMEM246, TNFRSF4, TNFSF10, TNNC2, TSSK3, USP50, VSIG8, WDR31, WFIKKN1, ZFHX2, ZFP14, ZFP37, ZNF583, ZNF750, ZNF883 |
| Rh1 | APCDD1, APOBEC3A_B, APOC1, ARIH2OS, ATF3, ATP5L2, ATP8B3, C11orf45, C11orf52, C11orf71, C14orf132, C15orf62, C17orf97, C1QTNF9B-AS1, C1R, C8orf4, CADM3, CAPS2, CCDC24, CD79A, CD79B, CECR6, CHN2, CLDN3, CMPK2, CPA3, CSF3, CYP26B1, CYTH4, DUSP1, EGR3, EPHX4, ESR2, FAM47E-STBD1, FCER1G, FMO4, FOS, FOSB, GDF9, GHRL, GNAT1, GNRH2, GPR84, GRIN2C, HCN3, HERC5, HGH1, HOXB9, HTR7, ID1, ID2, IFI30, IL13, IL17RE, IL18, IQCJ, ITGA2B, JAM2, KAZALD1, KCNE1L, KIAA1683, LAT2, LCT, LIN7B, LOC100288814, LOC100652758, LRG1, LSMEM1, MAFA, MAMDC2, MAP3K13, MIA, MMP28, MOBP, MT1F, MTMR8, MUC20, NCR1, NDUFA4L2, NEIL1, NPTX1, NR4A1, OIT3, OR1B1, OR1F1, OR1L8, OR2D2, OVOL3, OXTR, PARM1, PARVG, PCDHB13, PDK4, PEX11G, PIP5KL1, PPP1R36, PTK2B, RARRES3, RGN, RHBDL1, S100A14, S100A4, SDK1, SLC22A23, SLCO1C1, SNAI1, SOHLH2, SPAG4, SPINK5, ST20, TAP2, TAS1R3, TBR1, TBX6, TMEM121, TNFRSF11B, TNFRSF4, TNFSF13B, TSSK3, USP50, VIP, VSIG8, ZFHX2, ZKSCAN7, ZNF233, ZNF442 |

| **Supplementary Table 2.** Summary of GSEA results in VEGF-treated HUVECs  **A.** The positively scoring gene sets in VEGF-treated HUVECs | | | | |  |  |  |  |
| --- | --- | --- | --- | --- | --- | --- | --- | --- |
| NAME | NES | NOM p-val | FDR q-val | FWER p-val | RANK AT MAX | LEADING EDGE | SIZE | ES |
| TNFA_SIGNALING_VIA_NFKB | 5.74E+00 | 0 | 0 | 0 | 4448 | tags=70%, list=34%, signal=104% | 180 | 3.69E-01 |
| MYC_TARGETS_V1 | 4.72E+00 | 0 | 0 | 0 | 8748 | tags=94%, list=66%, signal=275% | 197 | 2.87E-01 |
| EPITHELIAL_MESENCHYMAL_TRANSITION | 3.75E+00 | 0 | 0 | 0 | 6072 | tags=71%, list=46%, signal=129% | 170 | 2.50E-01 |
| KRAS_SIGNALING_UP | 3.49E+00 | 0 | 0 | 0 | 1888 | tags=39%, list=14%, signal=45% | 143 | 2.52E-01 |
| OXIDATIVE_PHOSPHORYLATION | 3.18E+00 | 0 | 0 | 0 | 9949 | tags=94%, list=75%, signal=375% | 197 | 1.95E-01 |
| INFLAMMATORY_RESPONSE | 3.10E+00 | 0 | 0 | 0 | 2469 | tags=42%, list=19%, signal=51% | 129 | 2.34E-01 |
| MTORC1_SIGNALING | 3.02E+00 | 0 | 0 | 0 | 9111 | tags=88%, list=69%, signal=277% | 194 | 1.90E-01 |
| HYPOXIA | 2.68E+00 | 0 | 0 | 0 | 5776 | tags=60%, list=44%, signal=106% | 177 | 1.70E-01 |
| MYC_TARGETS_V2 | 2.65E+00 | 0 | 1.79E-04 | 1.00E-03 | 7037 | tags=82%, list=53%, signal=175% | 57 | 2.94E-01 |
| UNFOLDED_PROTEIN_RESPONSE | 2.61E+00 | 0 | 3.02E-04 | 2.00E-03 | 9224 | tags=91%, list=70%, signal=298% | 111 | 2.14E-01 |
| ANGIOGENESIS | 2.35E+00 | 0 | 3.10E-03 | 2.10E-02 | 6480 | tags=86%, list=49%, signal=168% | 28 | 3.68E-01 |
| PROTEIN_SECRETION | 2.28E+00 | 0 | 3.81E-03 | 2.70E-02 | 9181 | tags=89%, list=69%, signal=290% | 94 | 2.01E-01 |
| TGF_BETA_SIGNALING | 2.27E+00 | 0 | 3.77E-03 | 2.90E-02 | 7826 | tags=86%, list=59%, signal=210% | 51 | 2.72E-01 |
| IL2_STAT5_SIGNALING | 2.27E+00 | 1.92E-03 | 3.61E-03 | 3.00E-02 | 5249 | tags=55%, list=40%, signal=90% | 162 | 1.54E-01 |
| MITOTIC_SPINDLE | 2.10E+00 | 6.04E-03 | 6.94E-03 | 6.30E-02 | 10353 | tags=91%, list=78%, signal=412% | 198 | 1.28E-01 |
| ESTROGEN_RESPONSE_EARLY | 1.93E+00 | 8.00E-03 | 1.81E-02 | 1.63E-01 | 6398 | tags=62%, list=48%, signal=118% | 156 | 1.33E-01 |
| ADIPOGENESIS | 1.89E+00 | 4.10E-03 | 2.12E-02 | 2.02E-01 | 8898 | tags=79%, list=67%, signal=239% | 180 | 1.23E-01 |
| DNA_REPAIR | 1.82E+00 | 8.26E-03 | 3.14E-02 | 2.99E-01 | 8965 | tags=81%, list=68%, signal=249% | 142 | 1.34E-01 |
| ANDROGEN_RESPONSE | 1.62E+00 | 5.59E-02 | 8.73E-02 | 6.19E-01 | 8035 | tags=76%, list=61%, signal=191% | 90 | 1.49E-01 |
| REACTIVE_OXIGEN_SPECIES_PATHWAY | 1.60E+00 | 4.06E-02 | 9.08E-02 | 6.49E-01 | 8196 | tags=82%, list=62%, signal=215% | 45 | 2.03E-01 |
| UV_RESPONSE_DN | 1.59E+00 | 4.90E-02 | 8.88E-02 | 6.63E-01 | 6669 | tags=62%, list=50%, signal=124% | 135 | 1.19E-01 |
| APICAL_JUNCTION | 1.59E+00 | 4.85E-02 | 8.49E-02 | 6.64E-01 | 6351 | tags=59%, list=48%, signal=112% | 153 | 1.09E-01 |
| PI3K_AKT_MTOR_SIGNALING | 1.59E+00 | 4.07E-02 | 8.44E-02 | 6.78E-01 | 7078 | tags=67%, list=54%, signal=144% | 89 | 1.40E-01 |
| **B.** The negatively scoring gene sets in VEGF-treated HUVECs | | | | | | | | |
| NAME | NES | NOM p-val | FDR q-val | FWER p-val | RANK AT MAX | LEADING EDGE | SIZE | ES |
| E2F_TARGETS | -3.35E+00 | 0 | 0 | 0 | 8883 | tags=87%, list=67%, signal=262% | 198 | -2.05E-01 |
| G2M_CHECKPOINT | -3.05E+00 | 0 | 0.00015385 | 0.001 | 8928 | tags=86%, list=67%, signal=260% | 197 | -1.86E-01 |
| KRAS_SIGNALING_DN | -2.20E+00 | 0.004024145 | 0.00412503 | 0.04 | 3683 | tags=48%, list=28%, signal=66% | 85 | -2.05E-01 |
| FATTY_ACID_METABOLISM | -1.83E+00 | 0.013944224 | 0.02996195 | 0.306 | 8322 | tags=76%, list=63%, signal=204% | 135 | -1.35E-01 |
| SPERMATOGENESIS | -1.64E+00 | 0.026584867 | 0.05730253 | 0.578 | 4872 | tags=52%, list=37%, signal=81% | 83 | -1.51E-01 |

**Supplementary Table 3.** Primers used for real-time PCR

| Target gene | Primer sequence (5’-3’) | |
| --- | --- | --- |
|  | Forward | Reverse |
| *NR4A1* | ATGCCCTGTATCCAAGCCC | GTGTAGCCGTCCATGAAGGT |
| *NR4A2* | GTTCAGGCGCAGTATGGGTC | CTCCCGAAGAGTGGTAACTGT |
| *EGR1* | GGTCAGTGGCCTAGTGAGC | GTGCCGCTGAGTAAATGGGA |
| *NR4A3* | CATACAGCTCGGAATACACCAC | CCCTCCACGAAGGTACTGATG |
| *SOCS3* | CCTGCGCCTCAAGACCTTC | GTCACTGCGCTCCAGTAGAA |
| *PTGS2* | CTGGCGCTCAGCCATACAG | CGCACTTATACTGGTCAAATCCC |
| *SGK1* | GCAGAAGAAGTGTTCTATGCAGT | CCGCTCCGACATAATATGCTT |
| *F3* | GGCGCTTCAGGCACTACAA | TTGATTGACGGGTTTGGGTTC |
| *BHLHE40* | GACGGGGAATAAAGCGGAGC | CCGGTCACGTCTCTTTTTCTC |
| *CXCL1* | AACCGAAGTCATAGCCACAC | GTTGGATTTGTCACTGTTCAGC |
| *HBEGF* | ATCGTGGGGCTTCTCATGTTT | TTAGTCATGCCCAACTTCACTTT |
| *BMP2* | TTCGGCCTGAAACAGAGACC | CCTGAGTGCCTGCGATACAG |
| *PPAP2B* | TGAGAGCATCAAGTACCCACT | ACGTAGGGGTTCTGA ATCGTC |
| *TNFα* | CCTCTCTCTAATCAGCCCTCTG | GAGGACCTGGGAGTAGATGAG |
| *β-actin* | AGCACAATGAAGATCAAGAT | TGTAACGCAACTAAGTCATA |

**Supplementary Table 4.** Antibodies

| Antibody | Cat. No. | Manufacturer | Dilution |
| --- | --- | --- | --- |
| Primary |  |  |  |
| Rabbit anti-TNFα | CST-3707 | Cell Signaling | 1:1000 |
| Rabbit anti-phospho-NFκB p65 (Ser536) | CST-3033 | Cell Signaling | 1:1000 |
| Rabbit anti-NFκB p65 | CST-8242 | Cell Signaling | 1:1000 |
| Mouse anti-phospho-IκBα (Ser32/36) | CST-9246 | Cell Signaling | 1:1000 |
| Rabbit anti-IκBα | CST-9242 | Cell Signaling | 1:1000 |
| Mouse anti-β-actin | sc-47778 | Santa Cruz | 1:1000 |
| Secondary |  |  |  |
| HRP-linked anti-rabbit IgG | CST-7074 | Cell Signaling | 1:1000 |
| HRP-linked anti-mouse IgG | CST-7076 | Cell Signaling | 1:1000 |
